# Role of the Irr Protein in the Regulation of Iron Metabolism in *Rhodobacter sphaeroides*

**DOI:** 10.1371/journal.pone.0042231

**Published:** 2012-08-07

**Authors:** Verena Peuser, Bernhard Remes, Gabriele Klug

**Affiliations:** Institut für Mikrobiologie und Molekularbiologie, University of Giessen, Giessen, Germany; Newcastle University, United Kingdom

## Abstract

In Rhizobia the Irr protein is an important regulator for iron-dependent gene expression. We studied the role of the Irr homolog RSP_3179 in the photosynthetic alpha-proteobacterium *Rhodobacter sphaeroides*. While Irr had little effect on growth under iron-limiting or non-limiting conditions its deletion resulted in increased resistance to hydrogen peroxide and singlet oxygen. This correlates with an elevated expression of *katE* for catalase in the Irr mutant compared to the wild type under non-stress conditions. Transcriptome studies revealed that Irr affects the expression of genes for iron metabolism, but also has some influence on genes involved in stress response, citric acid cycle, oxidative phosphorylation, transport, and photosynthesis. Most genes showed higher expression levels in the wild type than in the mutant under normal growth conditions indicating an activator function of Irr. Irr was however not required to activate genes of the iron metabolism in response to iron limitation, which showed even stronger induction in the absence of Irr. This was also true for genes *mbfA* and *ccpA*, which were verified as direct targets for Irr. Our results suggest that in *R. sphaeroides* Irr diminishes the strong induction of genes for iron metabolism under iron starvation.

## Introduction

As cofactor of several enzymes and regulatory proteins iron is an essential element for living organisms. However, since reduced iron potentiates oxygen toxicity by the production of hydroxyl radicals in the Fenton reaction, life in the presence of oxygen requires a strict regulation of iron metabolism. Several regulators of iron metabolism have been investigated in bacteria. In *Escherichia coli* and in many other bacteria the Fur protein is a major regulator for iron-dependent gene expression (reviewed in e.g. [1]). Fur binds to DNA at a specific sequence (Fur box) [2] and acts as transcriptional repressor in the presence of iron. Iron regulation in alpha-proteobacteria mainly occurs by regulators different from this type of Fur protein, namely by Irr, RirA and IscR (reviewed in [3]). RirA and IscR both belong to the Rrf2 superfamily of transcriptional regulators. In *E. coli* IscR mainly regulates the Fe-S cluster biogenesis genes (*suf* genes) [4], [5]. To date its function in alpha-proteobacteria has not been investigated. RirA represses more than 80 transcriptional units in *Rhizobium* and *Sinorhizobium* in high iron conditions [6], [7]. Another Fur-like protein detected in alpha-proteobacteria is rather involved in the regulation of Mn^2+^ transport and was therefore designated “Mur” (manganese uptake regulator) [8], [9], [10], [11]. In *Rhodobacter sphaeroides* Mur has not only a role in manganese homeostasis but also affects regulation of iron metabolism [12].

An important function in regulating iron metabolism in Rhizobia could be attributed to the Irr protein (reviewed in [3]). Irr proteins are found in members of the Rhizobiales, Rhodobacterales and few other genera, and form a distinct sub-branch of the Fur superfamily [13]. In Rhizobiales most iron-dependent genes are regulated by Irr [14], [15], [16], [17]. In all species investigated to date Irr represses genes under iron depletion, which is opposite to the function of RirA. Irr binds to conserved sequences, the so-called Irr boxes or ICE motifs (iron control elements) close to the promoters of its target genes. At high iron concentrations Irr is degraded in *Bradyrhizobium japonicum* but not in *Rhizobium leguminosarum* [18]. In *B. japonicum* this degradation is mediated by its interaction with heme, whose intracellular concentration increases with external iron availability [19]. Heme can directly interact with the Irr protein but interaction is more efficient if heme is delivered by the heme synthesis enzyme ferrochelatase [20]. Reactive oxygen species (ROS) seem to promote the heme dependent degradation of Irr [21].

*R. sphaeroides* is a facultative photosynthetic bacterium, which can generate ATP by anoxygenic photosynthesis but can also generate ATP from aerobic or anaerobic respiration. At high oxygen concentration the formation of photosynthetic complexes is repressed. When the oxygen tension in the environment drops, photosynthesis genes are induced. Several proteins involved in redox-dependent gene regulation have been identified in *R. sphaeroides* [22], [23] and also the response to different ROS has been studied intensively [24], [25], [26], [27], [28], [29], [30], [31]. A transcriptome study revealed that many genes of iron metabolism are induced in response to hydrogen peroxide [30]. Only a few of these genes are under control of the intensively studied OxyR regulator [31]. Another transcriptome study revealed only very limited overlap of the response of *R. sphaeroides* to iron limitation and to oxidative stress [12]. The regulatory link between oxidative stress response and iron metabolism remains to be elucidated for alpha-proteobacteria.

A bioinformatic analysis based on experimental data from Rhizobia predicted iron responsive regulators and DNA target sequences for such regulators in alpha-proteobacteria including *Rhodobacter* [13] and suggested putative Irr (RSP_3179), Fur/Mur (RSP_2494), Fur/Zur (RSP_3569) and IscR (RSP_0443) regulators in *R. sphaeroides*. No RirA homolog is found in Rhodobacterales. A strain lacking the Fur/Mur protein was clearly impeded in growth by iron limitation indicating a role of Fur/Mur in regulation of iron metabolism. This was supported by transcriptome data [12].

Here we present an analysis of the role of the Irr protein (RSP_3179) on iron metabolism and resistance to oxidative stress in the photosynthetic alpha-proteobacterium *R. sphaeroides*.

## Results

### The Irr Protein of *R. sphaeroides* does not Influence Growth Under Iron Limitation but Resistance to Oxidative Stress

**Figure 1:**
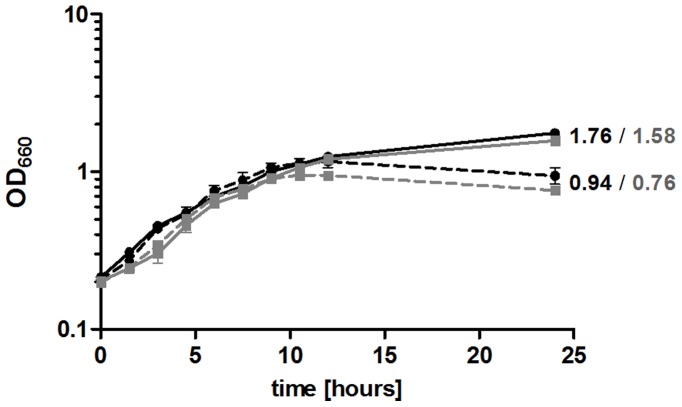
Growth curves of the *R. sphaeroides* wild type (black) and the 2.4.1Δ*irr* mutant (gray) under normal iron (continuous line) and under iron limitation (dashed line) conditions are shown. The optical density at 660 nm (OD_660_) of microaerobically grown *R. sphaeroides* cultures was determined over time. The data represent the mean of at least three independent experiments and error bars indicate standard error of the mean.

Exponential phase cultures of the *R. sphaeroides* wild type 2.4.1 and the 2.4.1Δ*irr* deletion strain were subjected to iron limitation as described previously [12]. *R. sphaeroides* was cultivated without adding external Fe(III)citrate to the malate minimal medium but with the iron chelator 2,2′-dipyridyl. Cultivation was performed under microaerobic conditions to exclude the possibility that change in expression of genes for iron metabolism is caused by oxidative stress as observed previously for *R. sphaeroides* [30]. Growth curves confirmed our previous observation [12] that the wild type stops growing at an earlier time point during transition to stationary phase when iron is limiting (Fig. 1). The growth of the 2.4.1Δ*irr* mutant (Table S4) was similar to that of the wild type. The mutant stopped growing a little bit earlier under iron limitation but this difference was not significant (Fig. 1). The *irr* deletion strain was clearly less impeded in growth by iron limitation than the 2.4.1Δ*fur*/*mur* mutant that was characterized previously [12]. It can be excluded that the growth phenotypes are due to dipyridyl toxicity because cultures grown under decreased iron availability without added iron chelator are also impaired in growth (data not shown). Excess amounts of iron (20 µM Fe(III)citrate) did not influence the growth behavior of *R. sphaeroides* (data not shown). Furthermore, the wild type and the *irr* deletion strain were tested for porphyrin accumulation during growth in iron-deficient media. Spectral analyses did not reveal detectable levels of protoporphyrin in supernatants of *R. sphaeroides* cultures grown under iron limitation (data not shown). These results suggest that, in contrast to studies in *Brad. japonicum* and *Brucella abortus*, *R. sphaeroides* Irr is not involved in the down-regulation of heme biosynthesis under iron limitation. This view is supported by real-time RT-PCR data for the *hemB* and *hemH* genes (Fig. S1). *R. sphaeroides* Irr lacks the heme regulatory motif (HRM) that is associated with binding to heme and the turnover of the protein in *Brad. japonicum*. However, as heme can interact with an Irr protein lacking this motif in *Bru. abortus* [32] and *R. leguminosarum* [18], binding of heme to *R. sphaeroides* Irr was analyzed. The absorption spectrum of heme in the presence and absence of purified recombinant Irr was recorded. The 388 nm absorption peak of heme shifted to 430 nm in the presence of Irr (Fig. S2), supporting that heme binds to *R. sphaeroides* Irr under the conditions tested. We also used the *R. sphaeroides* IscR protein, which was purified with the identical protocol in this assay and did not result in a shift of the heme absorbance.

**Figure 2:**
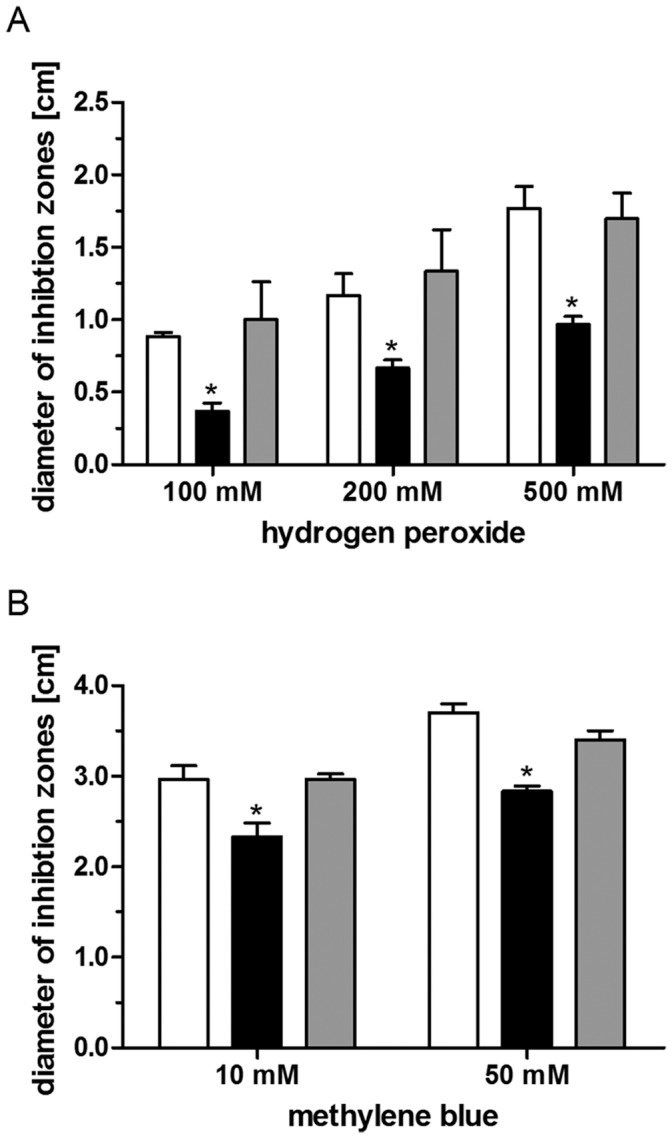
Sensitivity of *R. sphaeroides* wild type, 2.4.1Δ*irr* mutant and complemented mutant to (photo-) oxidative stress. Inhibition of growth of the wild type (white bars), the *irr* deletion mutant (black bars) and the complemented mutant (gray bars) to hydrogen peroxide (A) and methylene blue (B) as determined by inhibition zone assays. Each bar represents the mean of at least three independent experiments and error bars indicate standard deviation. Levels of significance are indicated as follows: *P≤0.01.

The sensitivity of the 2.4.1Δ*irr* mutant to hydrogen peroxide and singlet oxygen was tested by zone inhibition assays. Singlet oxygen was generated by applying methylene blue to the filter disks and illumination. For both types of ROS the inhibition zones were significantly smaller for the 2.4.1Δ*irr* mutant, indicating increased resistance (Fig. 2 A and B). When the *irr* gene was expressed in trans in strain 2.4.1Δ*irr* (pRK*irr*), a wild type-like phenotype could be restored (Fig. 2 A and B, gray bars).

Paraquat was used to generate superoxide stress. However, the inhibition zones were too diffuse for reliable quantification. Therefore growth was followed in liquid cultures containing 250 µM paraquat. No significant difference in growth between the wild type and the 2.4.1Δ*irr* mutant was observed (data not shown).

### Higher Resistance to ROS in the 2.4.1Δ*irr* Mutant Correlates with Elevated *katE* Expression Under Non-stress Conditions

**Figure 3:**
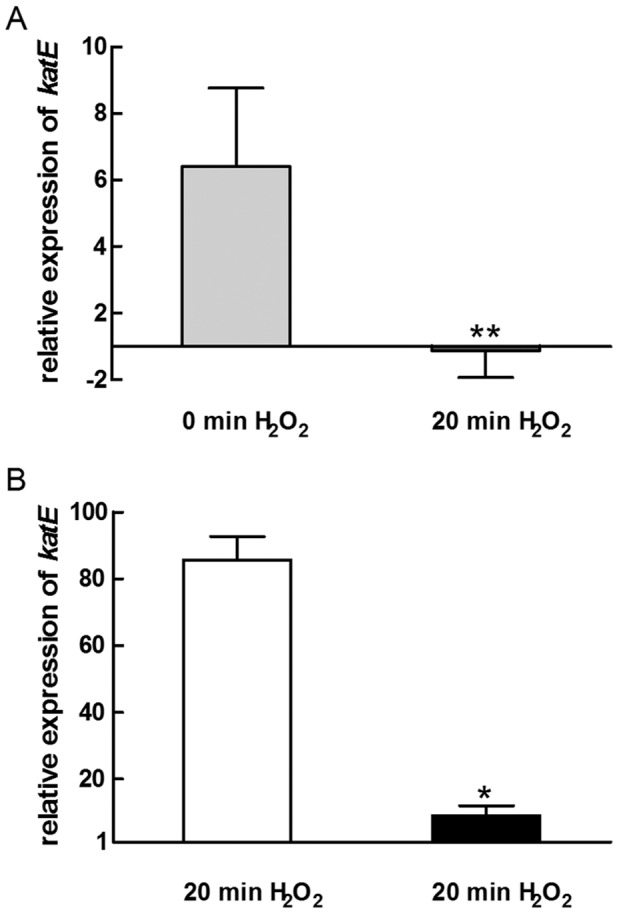
Relative expression of *katE* (RSP_2779) in *R. sphaeroides* wild type and 2.4.1Δ*irr* mutant. (A) Real-time RT-PCR was used to investigate the relative expression of *katE* in 2.4.1Δ*irr* mutant 0 min (light gray bar) and 20 min (dark gray bar) after exposure to 1 mM H_2_O_2_ compared to the wild type. (B) Relative *katE* expression 20 min of 1 mM H_2_O_2_ in the *R. sphaeroides* wild type (white bar) and the *irr* deletion mutant (black bar). Values were normalized to *rpoZ* and to the control at time point 0. The data represent the mean of three independent experiments and error bars indicate standard deviation. Levels of significance are indicated as follows: *P≤0.01; **P≤0.05.

Catalases make a major contribution to resistance against ROS since they rapidly detoxify hydrogen peroxide. In *R. sphaeroides* wild type *katE* expression is strongly induced within one minute after hydrogen peroxide addition, while *katC* does not respond to this stress [29]. The *katE* gene (RSP_2779) is under control of the OxyR regulator and a lack of OxyR leads to increased sensitivity to hydrogen peroxide [29]. Our microarray analyses did not reveal reliable data for *katE* expression (low *A* values). In order to see, whether the increased resistance of the 2.4.1Δ*irr* mutant correlates with *katE* expression, real-time RT-PCR was applied to compare *katE* expression levels to those of the wild type. When cultures were grown under microaerobic conditions (approx. 30 µM oxygen) without the addition of hydrogen peroxide, *katE* mRNA levels were about 6 fold higher in strain 2.4.1Δ*irr* compared to the wild type. 20 min after addition of hydrogen peroxide both strains showed almost identical *katE* expression levels (Fig. 3A). Fig. 3B shows the change in *katE* expression in both strains after addition of hydrogen peroxide. While *katE* mRNA levels in the wild type where about 80 fold increased 20 min after hydrogen peroxide addition compared to untreated cultures, *katE* levels were only slightly increased in the 2.4.1Δ*irr* mutant. We conclude that the mutant lacking the Irr protein has already very high *katE* levels under non-stress conditions, which are similar to the levels the wild type reaches only after hydrogen peroxide addition. Additionally, the expression of *bfd* (RSP_1547), RSP_1090 and *tonB* (RSP_0922) with and without added hydrogen peroxide was assessed using real-time RT-PCR. The behavior of these OxyR-dependent genes was similar to the expression pattern as described for *katE* (data not shown).

### Response of the Protein-coding Transcriptome of the 2.4.1Δ*irr* Mutant to Iron Limitation

We have previously characterized the response of the wild type transcriptome to iron limitation applying a high-density oligonucleotide microarray [12]. In the study presented here the effect of Irr on the transcriptome of *R. sphaeroides* was analyzed.

Under normal iron conditions, the abundance of 0.2% of the genes with a reliable *A* value (≥12) was changed by ≥1.75-fold and of 44.6% by ≤0.57-fold in the 2.4.1Δ*irr* mutant compared to wild type cells under the same conditions (Table S1). Under iron limitation, the abundance of 2.6% of the genes with a reliable *A* value was changed by ≥1.75-fold and of 14.6% by ≤0.57-fold in the 2.4.1Δ*irr* mutant compared to wild type cells under the same conditions (Table S1).

Under iron limitation, the abundance of 44.1% of those genes with a reliable *A* value was changed by ≥1.75-fold and of 0.6% by ≤0.57-fold in the 2.4.1Δ*irr* mutant compared to mutant cells under normal iron conditions (Table S1). Table 1 gives an overview on those genes, grouped to functional categories, that are differently expressed (≥1.75 or ≤0.57) in the mutant strain and also lists the changes previously determined for the wild type under iron limitation [12].

**Table 1:** Selection of iron-responsive genes in *R. sphaeroides.*

		Ratio					
Category and RSP no.	Gene	Δ*irr* +Fe vs. wild type +Fe^a^	Δ*irr* –Fe vs. wild type –Fe^a^		Δ*irr –*Fe vs. Δ*irr* +Fe^a^	wild type –Fe vs. wild type +Fe^b^	Description
**Iron uptake**							
RSP_0920	*exbB*	(0.66)	1.09		**14.82**	**4.29**	Biopolymer transport protein
RSP_0921	*exbD*	(0.56)	0.92		**14.98**	**3.49**	Biopolymer transport protein
RSP_0922		0.84	0.99		**3.70**	**2.36**	Putative TonB protein
RSP_1440		(0.75)	0.73		**7.19**	**3.92**	TonB dependent ferrisiderophore
RSP_1548	*irpA*	(0.56)	1.25		**19.66**	**4.05**	Iron-regulated protein
RSP_1818	*feoB*	**0.46**	1.01		**3.65**	1.36	Fe^2+^ transport system protein
RSP_1819	*feoA1*	**0.49**	1.11		**3.72**	1.55	Fe^2+^ transport system protein
RSP_2913		(0.46)	0.96		**13.21**	**3.22**	ABC Fe^3+^ siderophore transporter
RSP_3220		(0.50)	0.70		(7.35)	**1.91**	ABC ferric siderophore transporter
RSP_6006	*hemP*	(0.64)	1.27		**34.65**	**5.18**	Hemin uptake protein
RSP_6020	*feoA2*	**0.43**	0.86		**4.03**	1.28	Fe^2+^ transport system protein
RSP_7397		(0.31)	0.81		(4.85)	**1.79**	ABC Fe^3+^ siderophore transporter
**Iron storage**							
RSP_0352		**0.46**	0.67		**2.05**	1.15	Probable ferredoxin
RSP_0850	*mbfA*	**3.65**	**8.04**		**1.75**	(1.47)	Membrane-bound ferritin
RSP_1546	*bfr*	(0.55)	1.27		**4.52**	**1.99**	Bacterioferritin
RSP_1547	*bfd*	(0.49)	1.32		**12.31**	**2.71**	Bacterioferritin-associated ferredoxin
RSP_2424		0.67	0.77		**1.76**	1.51	Ferredoxin II
RSP_3342	*bfr*	**0.54**	0.70		0.90	1.01	Bacterioferritin
**Iron utilization**							
RSP_0434	*sufD*	1.48	**2.60**		**4.32**	**2.46**	Fe-S cluster assembly/repair
RSP_0437	*sufC*	1.46	**2.42**		**4.22**	**1.93**	Fe-S cluster assembly/repair
RSP_0439		1.50	**2.86**		**3.42**	**1.81**	Hypothetical protein
RSP_0440	*sufB*	1.72	**2.74**		**3.69**	1.63	Fe-S cluster assembly/repair
RSP_0442		(0.74)	1.39		(3.92)	1.56	Putative aminotransferase
RSP_0443		(0.62)	1.34		(4.67)	**1.77**	Rrf2 family transcriptional regulator
RSP_2395	*ccpA*	0.90	**1.80**		**2.32**	0.78	BCCP, cytochrome c peroxidase
**Stress response**							
RSP_0166	*dksA*	**0.51**	0.99		1.63	1.12	DnaK suppressor protein
RSP_0697		**0.43**	1.17		**1.81**	0.75	Universal stress protein
RSP_1172	*dnaJ*	**0.50**	0.68		1.40	1.09	Chaperone
RSP_1194	*grxC*	**0.54**	0.96		1.55	1.05	Glutaredoxin
RSP_1219	*grpE*	**0.57**	0.77		1.74	1.11	Putative chaperone protein GrpE
RSP_1529	*trxA*	**0.56**	0.82		1.70	1.07	Thioredoxin
RSP_1572		**0.55**	1.47		1.52	0.73	Heat shock protein. Hsp20 family
RSP_2310	*groES*	0.72	0.82		**2.31**	1.49	Chaperonin Cpn10 (GroES) (protein folding)
RSP_2311	*groEL*	**0.60**	0.66		**2.41**	1.21	Chaperonin GroEL
RSP_2654		0.58	0.68		**1.90**	1.59	DnaK suppressor protein
RSP_2693		(0.42)	0.67		**2.45**	1.62	Superoxide dismutase (Fe-Mn)
RSP_2843	*hfq*	**0.50**	0.81		**1.77**	1.22	RNA-binding protein Hfq
RSP_4203		**0.39**	1.03		**2.31**	1.05	putative glutaredoxin family protein/Thio-disulfide isomerase
**Oxidative phosphorylation**							
RSP_0100–0104	*nuo*	(0.44)–**0.56**	0.83–1.15		1.21**–1.86**	0.90–1.05	Putative NADH dehydrogenase
RSP_1035–39	*atp*	**0.45–0.54**	**0.43**–0.62		**1.89–2.41**	1.23–**1.75**	F_0_F_1_ ATP synthase
RSP_2296–2300	*atp*	**0.48–0.56**	**0.56**–0.66		**1.90–2.25**	1.35–1.65	ATP synthase
RSP_2512–30	*nuo*	**0.44–0.57**	0.58–0.76		**1.87–2.35**	1.30–1.60	NADH dehydrogenase
**Transporter**							
RSP_0371		**0.50**	0.88		**1.84**	1.10	ABC basic amino acid transporter
RSP_0372		**0.55**	0.75		1.55	1.18	ABC basic amino acid transporter
RSP_0910–12	*dct*	**0.52**–0.62	**0.41**–**0.51**		**1.85–2.23**	1.71–**1.90**	TRAP-T family transporter
RSP_1747	*bztA*	**0.47**	0.83		**2.02**	1.03	ABC glutamate/glutamine/aspartate/asparagines transporter
RSP_1804	*ccmD*	**0.53**	1.00		1.72	1.08	Heme exporter protein D
RSP_2399		**0.51**	0.71		1.73	1.23	ABC putrescine transporter
RSP_2400		**0.49**	0.64		1.74	1.36	ABC putrescine transporter
RSP_3571	*znuA*	(2.98)	0.93		(0.26)	(1.62)	ABC zinc transporter
**Photo-synthesis**							
RSP_0261–63	*bch*	(1.17)–1.38	**2.35**–**2.73**		**0.48–0.53**	0.67–0.85	Chlorophyllide reductase
RSP_0277	*bchP*	0.95	**1.81**		0.88	0.95	Geranylgeranyl hydrogenase
RSP_0279	*bchG*	0.71	**1.81**		0.95	0.65	bacteriochlorophyll a synthase
RSP_0314	*pucB*	1.30	**3.16**		**0.51**	**0.57**	LHII beta, light-harvesting B800/850 protein
RSP_0315	*pucC*	0.97	2.57		0.60	(0.89)	Light-harvesting 1 (B870) complex assembly
RSP_0317	*hemN*	**0.38**	0.83		**2.10**	0.94	Coproporphyrinogen III oxidase
RSP_6256	*pucA*	1.14	**3.12**		0.64	**0.45**	LHII alpha, light-harvesting B800/850 protein
RSP_0679	*hemC*	**0.57**	1.07		1.37	0.92	Porphobilinogen deaminase
RSP_0680	*hemE*	**0.57**	0.93		1.48	1.05	Uroporphyrinogen decarboxylase
RSP_0693–96	*cco*	**0.44–0.47**	0.63–0.66		1.53–**2.21**	1.03–1.22	Cbb 3-type cytochrome c oxidase
RSP_0699	*hemZ*	**0.56**	0.75		**1.84**	1.64	Coproporphyrinogen III oxidase
RSP_1556	*puc2B*	1.23	**2.75**		**0.60**	0.68	Light-harvesting complex, beta subunit
RSP_6158	*puc2A*	1.08	**2.34**		0.70	**0.56**	Light-harvesting complex, alpha subunit

a Significant changes are in bold. Numbers in parentheses failed to meet the set *A* value criteria, while plain numbers show a lower fold change than ≥1.75 or ≤0.57. Selected genes that missed the cut-offs are included in this table to fully represent functional groups discussed.

b Values are taken from Peuser and colleagues (2011).

When comparing expression in the wild type and the mutant and the response to iron limitation three types of expression patterns can be discriminated. Genes of group I show lower expression in the mutant than in the wild type under normal growth conditions. Iron limitation results in stronger induction in the mutant and consequently expression levels are similar in both strains under iron limitation. For group II genes expression in the mutant is higher than in the wild type in presence or absence of iron and group III genes behave oppositely (lower expression in mutant under both conditions).

Most genes with predicted function in iron uptake or iron storage fall into group I. Genes for systems involved in the uptake of iron include e.g. *exbBD* and *tonB* (RSP_0920–22, 3.7–15.0), *hemP* (RSP_6006, 34.7), a gene encoding an iron-regulated protein (RSP_1548, 19.7), and genes for Fe^3+^-siderophore transporters (RSP_2913, 13.2; RSP_1440, 7.2). The genes RSP_3220 (7.4) and RSP_7397 (4.9) encoding subunits of siderophore transporter were also up-regulated in response to iron limitation, but did not pass our filtering criteria. The expression of iron storage genes encoding bacterioferritin *bfr* (RSP_1546, 4.5), bacterioferritin-associated ferredoxin *bfd* (RSP_1547, 12.3) and ferredoxin (RSP_0352, 2.1; RSP_2424, 1.8) was also increased under iron limitation.

Genes involved in stress responses or proteolysis also showed the group I expression pattern. They were weakly induced in the mutant upon iron limitation, while induction in the wild type was even less or not observed. As a consequence expression levels in both strains were similar under iron limitation. Genes with function in stress responses include e.g. genes encoding the DnaK suppressor protein (RSP_0166, RSP_2654), a universal stress protein (RSP_0697), chaperones like *grpE*, *groES*, *groEL* and *hfq* (RSP_1219, RSP_2310-11, RSP_2843), the superoxide dismutase encoding gene RSP_2693, and glutaredoxin and thioredoxin encoding genes.

In contrast to other genes of iron storage, *mbfA* showed a different expression pattern, that it shares with the *suf* genes (RSP_0434–0443) which are involved in *de novo* assembly and/or repair of iron-sulfur clusters (group II): expression levels in the mutant are higher than in the wild type in presence or absence of iron. *mbfA* (RSP_0850) encodes a membrane-bound ferritin and showed about 8 fold higher expression in the mutant compared to the wild type under iron limiting conditions. This results from 3 fold higher expression levels under normal growth and stronger induction in the mutant strain in response to iron limitation (factor 1.8). Some *suf* genes showed slightly higher expression in the mutant compared to wild type under normal growth and more than two-fold higher expression under iron limitation due to the stronger induction.

Most of the selected iron-responsive genes follow the group III expression pattern. Genes for energy metabolism (glycolysis, citric acid cycle, oxidative phosphorylation), fatty acid metabolism, some genes for transporters, genes involved in transcription, RNA processing, amino acid metabolism and translation or chemotaxis showed lower expression levels in the mutant strain compared to the wild type in the presence and absence of iron and showed slightly increased expression in response to iron limitation in the mutant (Table 1 and Table S2).

Interestingly, the ABC zinc transporter gene *znuA* (RSP_3571) showed a different expression pattern. Its expression was about three times higher in the *irr* mutant compared to the wild type under normal iron conditions and did not change in the presence of iron in the mutant strain compared to the wild type. All three types of expression patterns (group I, II or III) can be observed among the genes with a role in photosynthesis (Table 1 and Table S2).

**Figure 4:**
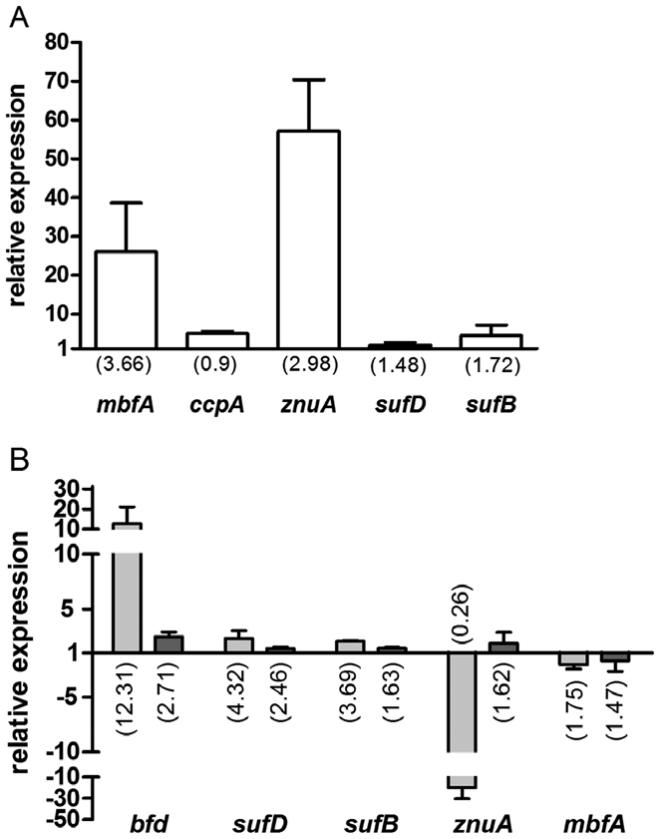
Validation of microarray data by real-time RT-PCR. Relative gene expression (A) in 2.4.1Δ*irr* under normal iron conditions compared to the wild type under normal iron conditions and (B) in 2.4.1Δ*irr* under iron limitation compared to normal iron conditions (light gray bars) and in wild type under iron limitation compared to normal iron conditions (dark gray bars). Values were normalized to *rpoZ* and to the respective control treatment. The data represent the mean of at least three independent experiments and error bars indicate standard deviation. Numbers in parentheses show the fold change of the respective genes as determined by microarray analysis.

To validate the microarray data real-time RT-PCR was used for the quantification of mRNAs transcribed from some selected genes. On the one hand the ratio of the expression in the 2.4.1Δ*irr* mutant was compared to that of the wild type strain under normal iron conditions (Fig. 4A), on the other hand the ratios of mRNA levels for the mutant strain grown in normal medium or under iron limitation were determined (Fig. 4B). Fig. 4B also includes the respective relative expression values of the wild type. Increase or decrease of expression levels as revealed by microarrays were confirmed by real-time RT-PCR, the extent of change was however different for some genes, mostly larger in the real-time RT-PCR data set.

In addition to protein-coding genes, the expression of intergenic regions (IGR) and small non-coding RNAs (sRNA) was analyzed as they can contribute to iron metabolism regulation as well. Northern Blot analysis was used to validate the expression levels as determined by the microarray approach. A selection of five sRNAs that showed altered expression in the microarray data in 2.4.1Δ*irr* mutant under iron limitation was used for Northern hybridization (Fig. S3). The abundance of RSs0827 and RSs0680a were increased in the wild type and in the mutant under iron limitation (2.4 fold and 2.5 fold, respectively) as confirmed by Northern blot analysis (2.9 fold and 2.1 fold, respectively). Northern blots revealed that the abundance of RSs0682 and RSs2978 was not altered in the *irr* deletion strain under iron starvation (0.9 fold and 1.4 fold, respectively), although microarray analysis gave a ratio of about 4 fold and 2 fold, respectively. RSs2430 showed a higher expression in the mutant under iron-limiting conditions in the microarray analysis (about 3 fold), which could not be confirmed by Northern Blot analysis (0.4 fold). All five sRNAs showed a similar pattern in the wild type under iron limitation compared to the mutant (Fig. S3). Thus, it was only possible to confirm the microarray data of sRNAs in parts. It is conceivable, that the discrepancy between both techniques is due to mis-hybridization on the chip. In general, sRNAs have a size between 50 and 250 nt. Consequently, in many cases only one or two 60nt-oligonucleotides per sRNA or IGR could be designed for microarray analysis.

### Irr Binds to Target Sequences in the Promoter Regions of the *mbfA* and *ccpA* Genes

Rodionov *et al.* (2006) predicted binding sites for the Irr protein in the upstream regions of the *R. sphaeroides* genes *mbfA* (RSP_0850) and *ccpA* (RSP_2395). *MbfA* encodes a membrane- bound ferritin, *ccpA* encodes a cytochrome c peroxidase. Iron limitation resulted in weak up-regulation of *mbfA* (about 1.5 fold) and weak down-regulation (factor 0.8) of *ccpA* in the wild type [12]. *MbfA* was strongly up-regulated (about 9 fold) by hydrogen peroxide stress, while *ccpA* was significantly down-regulated (about 5 fold) [30]. In the *irr* deletion mutant *mbfA* shows about 8 fold higher expression in comparison to the wild type under iron limitation and it is up-regulated by iron limitation (factor 1.75). The expression of *ccpA* is increased in 2.4.1Δ*irr* under iron limitation (about twofold) and its expression level is also higher in the mutant compared to the wild type under iron limitation (factor 1.8) (Table 1).

**Figure 5:**
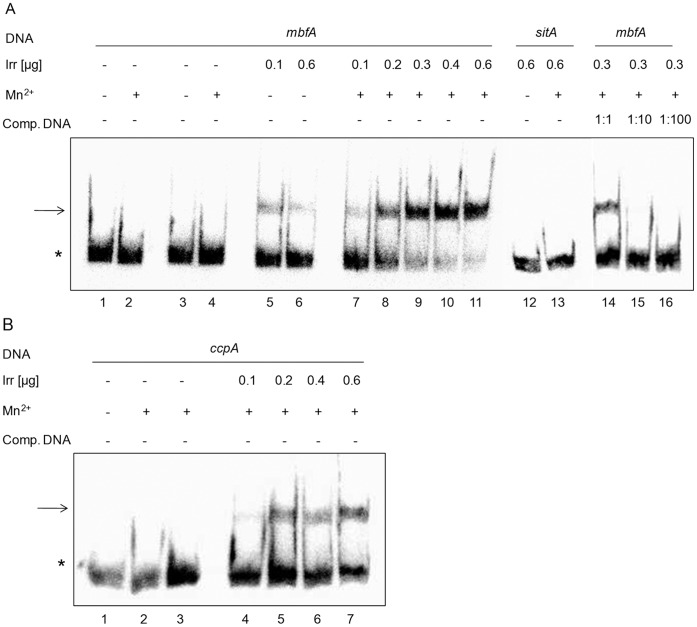
Binding of purified Irr to the promoter of *mbfA* and *ccpA* as determined by Electrophoretic Mobility Shift Assays. (A) Binding of Irr to the promoter region of *mbfA* (180 bp). All reactions contain the same amount of ^32^P end-labeled DNA fragment (3.08 fmol/lane) comprising the promoter sequence. Lanes 1–4 contain no Irr; lanes 3 and 4 contain 0.6 µg BSA; lanes 5 and 7 contain 0.1 µg Irr; lane 6 and 11–13 contain 0.6 µg Irr; lane 8 contains 0.2 µg Irr; lanes 9 and 14–16 contain 0.3 µg Irr; lane 10 contains 0.4 µg. Reactions contain 1 mM MnCl_2_ as indicated. Lanes 14–16 contain non-labeled DNA fragment *mbfA* in excess amount as cold competitor. Lanes 12 and 13 contain radioactively labeled *sitA* DNA fragment (180 bp) as unspecific DNA. (B) Binding of Irr to the promoter region of *ccpA* (168 bp). All reactions contain the same amount of ^32^P end-labeled DNA fragment (3.68 fmol/lane) comprising the promoter sequence. Lanes 1–3 contain no Irr; lane 3 contains 0.6 µg BSA; lane 4 contains 0.1 µg Irr; lane 5 contains 0.2 µg Irr; lane 6 contains 0.4 µg Irr; lane 7 contains 0.6 µg Irr. Reactions contain 1 mM MnCl_2_ as indicated. All reactions contain 1 µg of salmon sperm DNA as unspecific competitor. The asterisks and arrows show the location of free and Irr-bound ^32^P end-labeled DNA fragments, respectively.

To verify the binding of Irr to the upstream regions of *mbfA* and *ccpA* Irr was purified after heterologous overexpression and suitable DNA fragments were amplified for gel retardation analyses. For both fragments the formation of a retarded DNA protein complex was observed (Fig. 5 A+B). The presence of manganese favored formation of the Irr complexes. Molar excess of unlabeled DNA fragment decreased the amount of labeled DNA in the complex (Fig. 5A lanes 14–16 and data not shown), while the presence of molar excess of unspecific DNA (salmon sperm DNA) did not compete with complex formation. From the binding curves dissociation constants of 0.8±0.15 µM for *mbfA* and 0.9±0.1 µM for *ccpA*, respectively, were determined (Fig. S4). The Irr protein did not bind to a DNA fragment from the upstream region of *sit*A (Fig. 5 A lanes 12 and 13), which was predicted as a target for the Mur protein in *R. sphaeroides*. Furthermore, Figure S5 shows that the Irr protein neither binds to the upstream region of *katE* nor to the upstream region of *iscR* (RSP_0443). Taken together, these analyses confirmed a specific interaction of Irr and the *mbfA* and *ccpA* upstream regions.

**Figure 6:**
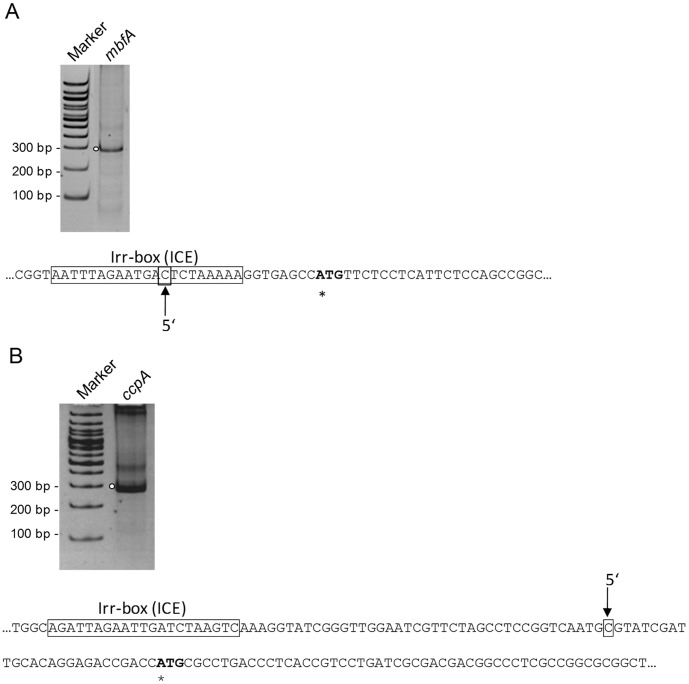
Determination of 5′ ends of *mbfA* (RSP_0850) (A) and *ccpA* (RSP_2395) (B) mRNA by 5′ rapid amplification of cDNA ends (RACE). Separation of 5′-RACE products *mbfA* and *ccpA* obtained from RNA extracts of the wild type strain under normal iron conditions. PCR products obtained after second PCR (nested) were separated on a 10% polyacrylamid gel and stained with ethidium bromide. Determined 5′ ends are indicated by an arrow. The putative translational start is indicated by an asterisk. The Irr-box (ICE, iron control element) is marked by a frame.

To verify that the Irr binding sites are located in an appropriate distance from the transcriptional start, we performed 5′ RACE to determine 5′ends of the *mbfA* and *ccpA* mRNAs. As indicated in Figure 6 5′ends were identified downstream of the Irr-box for *ccpA* (Fig. 6 B), while for *mbfA* the 5′end mapped within the Irr-box (Fig. 6 A).

## Discussion

The Irr protein was identified as an important regulator of iron metabolism in several alpha-proteobacteria. A mutant of *R. sphaeroides* lacking the predicted Irr homolog has a similar growth phenotype like the wild type under normal iron and iron limitation conditions. This suggests that Irr has no major function in the adaptation to growth under iron-limiting conditions. This was also observed for a mutant of *Bru. abortus* lacking Irr [32], while deletion of the *irr* gene in *Agrobacterium tumefaciens* diminished growth under iron limitation [33]. Furthermore, *irr* expression was not affected by the presence of iron in the *R. sphaeroides* wild type. This was also reported for *irr* expression of *Bru. abortus* [32]. Irr mutants of *Brad. japonicum, Rhizobium leguminosarum* and *Bru. abortus* accumulate protoporphyrin IX under iron limitation [11], [19], [32]. However, no protoporphyrin accumulation was observed in the *R. sphaeroides irr* deletion strain under iron-limiting conditions. Unlike the Rhizobiales species, *R. sphaeroides* requires protoporphyrin also as precursor for bacteriochlorophyll synthesis. The *Brad. japonicum* and the *Bru. abortus* Irr bind heme, although the latter does not contain the heme regulatory motif (HRM) and conserves only two histidines (HXH) of the second heme binding motif [34]. The HXH motif is also found in *R. sphaeroides* Irr and heme bound to the recombinant protein. In *R. sphaeroides* Irr and Irr proteins classified to the same branch [13] a proline is positioned between the two histidines, whereas in *Bru. abortus* Irr glutamine is the middle amino acid of the heme binding motif. No other Irr proten with a HPH motif has been analyzed in regard to heme binding up to now. Irr was identified in *Brad. japonicum* as a transcriptional repressor of *hemB* (5-aminolevulinate dehydratase) in the heme biosynthetic pathway [19]. However, *hemB* expression was not changed in 2.4.1Δ*irr* under iron limitation.

The view that Irr has no major function in iron regulation in *R. sphaeroides* is supported by a transcriptome analysis of the mutant strain. Many genes in strain 2.4.1Δ*irr* showed a lower expression level compared to the wild type in presence and absence of iron. The difference was mostly in the range of a factor of two and the affected genes fall into several different functional categories. It is highly unlikely that Irr would directly target all these genes, especially since no target sites are predicted [13]. Expression of *katE* and other OxyR-dependent genes was higher in the mutant compared to the wild type under non-stress conditions. Since activation of OxyR depends on oxidative stress this is indicative of a higher level of oxidative stress in the mutant. We hypothesize that the lower expression level of many genes in the mutant is most likely due to this increased oxidative stress in strain 2.4.1. Δ*irr*. The expression pattern of genes in the Irr mutant under non-stress conditions is however partly different from the effect of hydrogen peroxide on gene expression in *R. sphaeroides* [30]. The higher resistance of the Irr mutant to oxidative stress is also in agreement with the higher expression levels of genes for the oxidative stress response. Thus, the cells are prepared to counteract oxidative stress better than cells, in which the oxidative stress response still needs to be mounted.

All genes for iron metabolism that we reported before to be induced in response to iron limitation in *R. sphaeroides* wild type [12] are also induced in strain 2.4.1Δ*irr*. Indeed the induction in the Irr mutant was mostly stronger than in the wild type. Thus, Irr prevents even stronger induction of genes in response to iron starvation in the wild type.

Our study also identified two sRNAs, which are induced upon iron limitation. Irr had no influence on the expression of RSs0827, but expression levels of RSs0680a were higher in the absence of Irr. While the function of RSs0827 is unknown, RSs0680a is induced in response to singlet oxygen and even more in response to superoxide [25]. Thus, increased expression of RSs0680a in the mutant strain is another hint to increased levels of oxidative stress in the absence of Irr. Whether these sRNAs have a role in regulation of iron metabolisms as reported for sRNAs in other species [35], [36], [37] needs to be analyzed in the future.

Among the genes with reliable *A* value only *mbfA* (RSP_0850) encoding a membrane-bound ferritin showed significantly higher expression in the *irr* mutant compared to the wild type under normal iron and iron limitation conditions. In addition the *znuA* gene (RSP_3571) for an ABC zinc transporter showed higher expression in the mutant under normal iron conditions compared to the wild type. Expression levels where just below the *A* value but real-time RT-PCR confirmed the higher expression level in strain 2.4.1.Δ*irr* (Fig. 4 A). However, no Irr-box (also named ICE for iron control element) was predicted in the promoter region of *znuA* [13]. *MbfA* is one of the two genes of *R. sphaeroides* for which an Irr-box was predicted [13]. A putative transcriptional start site for the *mbfA* gene was determined within the Irr-box motif. This position of the Irr binding site in relation to the transcriptional start is in agreement with a repressor function of Irr as also demonstrated for e.g. the bll6680 (*bfr*, bacterioferritin) and blr7895 (rubrerythrin-like protein) genes in *Brad. japonicum* [14]. The affinity of Irr to these ICE motifs in *Brad. japonicum* was very high with K_Ds_ of 7 to 19 nM [15]. The K_D_ of the *R. sphaeroides* Irr protein to *mbfA* as determined by electrophoretic mobility shifts was about 800 nM, indicating a much lower *in vitro* affinity. The *mbfA* gene was only weakly induced by iron starvation in the Irr mutant (1.8 fold), similarly as in the wild type (about 1.5 fold). Thus, Irr has no major role in regulation of iron-dependent expression of *mbfA*.

The only other gene of *R. sphaeroides* for which an Irr-box in the promoter region was predicted [13] is *ccpA* (RSP_2395) encoding a cytochrome c peroxidase. This heme-iron protein reduces peroxides, which are generated by oxidative stress. Its expression level was similar in the wild type and the mutant under normal iron conditions but was slightly increased in the mutant compared to the wild type under iron limitation. Iron limitation resulted in weak induction of *ccpA* in the *irr* mutant, while it had no significant effect on *ccpA* expression in the wild type under the same conditions. We demonstrated *in vitro* binding of Irr to the *ccpA* promoter region with similar affinity as to the *mbfA* promoter region. The putative transcriptional start site mapped around 40 nt downstream of the Irr-box. In *Brad. japonicum* 17 iron-regulated genes with a putative Irr-box and a total of 172 ICE-like motifs were identified [14]. This search applied a more variable motif than the search by Rodionov *et al.* (2006), which predicted 23 Irr binding sites for *Brad. japonicum* and only 2 for *R. sphaeroides*. *in vitro* binding of Irr to these motifs was demonstrated for blr7895 (rubrerythrin-like protein) and for bll6680 (*bfr*) [14]. Only for few of the *Brad*. *japonicum* iron-regulated genes with a putative Irr binding site homologs with good similarity are found in *R*. *sphaeroides* (e.g. *acnA*, *leuC*, *lguL*, *fumC*). None of these genes showed a significant response to iron in *R. sphaeroides*. Our data imply different roles of Irr in gene regulation in individual alpha-proteobacteria. Table S3 summarizes the different features of Irr and Irr mutants in *R. sphaeroides* and the Rhizobiales species, which have been investigated in this regard.

Like the Irr mutants of *Bru. abortus* [38] and *A. tumefaciens* [33] the *R. sphaeroides* mutant showed higher resistance to oxidative stress, which is in agreement with increased *katE* expression under normal growth conditions. Interestingly both genes with Irr-boxes in *R. sphaeroides, mbfA* and *ccpA* have a function in oxidative stress defense. *MbfA* (RSP_0850) shares good homology with blr7895 (rubrerythrin-like protein) and showed an increased expression in the *irr* deletion strain compared to the wild type under non-stress conditions. Rubrerythrin is a structurally and biophysically well-characterized non-heme iron protein [39], [40]. It is hypothesized that rubrerythrin provides oxidative stress protection via catalytic reduction of intracellular hydrogen peroxide [41], [42], [43], although this function was disputed [44]. Since iron limitation causes oxidative stress due to decreased levels of iron-sulfur proteins with important roles in oxidative stress defense increased *katE* and maybe also *mbfA* and *ccpA* expression levels help to counteract this oxidative stress. A direct effect of Irr on *katE* expression is unlikely since no Irr-box is present in the *katE* promoter region and no *in vitro* binding was observed by gel shift experiments. Alternatively, the lack of Irr, which causes an enhanced induction of genes for iron uptake in response to iron starvation may cause the generation of ROS, which consequently activate *katE* expression and cause lower expression of many other genes. It is conceivable that under microaerobic growth a limitation of the up-regulation of genes in response to iron starvation is the biological function of Irr in *R. sphaeroides.* It would thus contribute to a balance between increase of iron-uptake systems, which counteracts iron limitation and the formation of ROS by too much iron import.

## Materials and Methods

### Bacterial Strains, Growth Conditions and Iron Limitation

Strains and plasmids used in this study are listed in Table S4.

*R. sphaeroides* strains were cultivated at 32°C in 50 ml Erlenmeyer flasks containing 40 ml malate minimal medium [45] with continuous shaking at 140 rpm. This growth is designated as microaerobic growth. At the chosen growth conditions the cultures contain about 2% oxygen (approx. 30 µM oxygen).

Conditions of iron limitation were achieved by cultivating *R. sphaeroides* without adding external Fe(III)citrate to the growth medium but with the iron chelator 2,2′-dipyridyl (30 µM; Merck). A small aliquot of cells were transferred from normal cultivation medium to iron-limited medium. The cells were grown overnight and then transferred two times more into iron-limited medium. Inductively coupled plasma mass spectrometry (ICP-MS) using Agilent 7500ce confirmed that the iron content was drastically reduced in iron-limited medium (from 140 µg/l to 16 µg/l) [46]. For gene expression studies cells were harvested at an OD_660_ of 0.4.

When required antibiotics were added to the liquid or solid growth medium at the following concentrations: kanamycin, 25 µg ml^−1^; tetracycline 2 µg ml^−1^ (for *R. sphaeroides*); and ampicillin, 200 µg ml^−1^; kanamycin, 25 µg ml^−1^; tetracycline, 20 µg ml^−1^ (for *E. coli*).

### Construction of an *R. sphaeroides irr* Deletion Mutant

*R. sphaeroides* strain 2.4.1Δ*irr* was generated by transferring the suicide plasmid pPHU2.4.1Δ*irr*::Km into *R. sphaeroides* 2.4.1, and screening for insertion of the kanamycin resistance cassette into the chromosome by homologous recombination. Briefly, parts of the *irr* gene (RSP_3179) of *R. sphaeroides* 2.4.1, together with upstream and downstream sequences, were amplified by PCR using oligonucleotides KO3179-Eco_A1 (5′-CGA AGC GAA TTC CCT GCC AGC C-3′), KO3179-Pst_A2 (5′-GAT TGC CGA TCG CTG CAG CAT TCC-3′) and KO3179-Pst_B1.3 (5′- CGA CAA CCA TCT GCA GTT CTA CTG GG -3′), KO3179-Pae_B2.3 (5′- GGC AGT TCC GCA TGC GGG ATC TCG -3′).

The amplified PCR fragments were cloned into the EcoRI-PstI and PstI-PaeI sites of the suicide plasmid pPHU281, generating the plasmid pPHU2.4.1Δ*irr*. A 1.3 kb PstI fragment containing the kanamycin cassette from pUC4K [47] was inserted into the PstI site of pPHU2.4.1Δ*irr* to generate pPHU2.4.1Δ*irr*::Km. This plasmid was transferred into *E. coli* strain S17-1 and diparentally conjugated into *R. sphaeroides* 2.4.1 wild type strain. Conjugants were selected on malate minimal salt agar plates containing 25 µg kanamycin ml^–1^. By insertion of the kanamycin cassette, 285 bp of the 441 bp *R. sphaeroides irr* gene (RSP_3179) was deleted. PCR analysis of chromosomal DNA was carried out to confirm the double crossover event of the kanamycin cassette into the *R. sphaeroides* chromosome (Fig. S6).

### Complementation of the *R. sphaeroides* Deletion Mutant 2.4.1Δ*irr*

For complementation of the *irr* deletion mutant of *R. sphaeroides* a 539 bp PCR fragment containing the entire *irr* gene (RSP_3179) along with 57 bp of the upstream and 49 bp of the downstream sequence of the *irr* gene was amplified by using the oligonucleotides 3179compl_fwd (5′-GCC GTC TAG AAA ACA TGG GTC TTT C-3′) and 3179compl_rev (5′-CTG CCC GCA GAA TTC GCA GAC G- 3′). Following digestion with XbaI and EcoRI, the fragment was cloned into the corresponding sites of pRK415, resulting in plasmid pRK2.4.1*irr*. To complement the *irr* deletion in the wild type strain 2.4.1, the plasmid pRK2.4.1*irr* was transferred into *E. coli* S17-1 and conjugated into the 2.4.1Δ*irr* strain by diparental conjugation.

### Inhibition Zone Assays

For inhibition zone assays cultures were grown microaerobically overnight at 32°C and then diluted to an OD_660_ of 0.2. Cultures were grown to an OD_660_ of 0.4 and 200 µl of the culture were mixed with 5 ml prewarmed top agar (0.8% (w/v) agar) and layered onto malate minimal salt medium plates. A 0.55 cm filter disk, containing 5 µl of hydrogen peroxide (100, 200 and 500 mM), was placed on the hardened top agar. Zones of inhibition were measured after incubation for 72 h at 32°C in the dark. Inhibition zone assays were also performed under a fluorescent tube (model NL 36 W/860 daylight) with filter disks containing 5 µl of 10 and 50 mM methylene blue to generate singlet oxygen. The assays were performed at least three times.

### Extraction of RNA and Quantitative Real-time RT-PCR

Cell samples from growth experiments (OD_660_ 0.4) were rapidly cooled on ice and harvested by centrifugation at 10 000 *g* in a cooled centrifuge. Total RNA was isolated by the peqGOLD TriFast ™ Kit (Peqlab) as described by the manufactures protocol. Samples were treated with 1 unit of RNase-free DNase I (Invitrogen) per 1 µg RNA to remove contaminating DNA. After DNase I treatment, the RNA was purified by standard procedures using a mixture of phenol/chloroform/isoamyl alcohol and chloroform/isoamyl alcohol before precipitating with sodium acetate and isopropanol. Contamination with remaining DNA was checked by PCR amplification of RNA samples using primers targeting *gloB* (RSP_0799-A: 5′-GAA CAA TTA CGC CTT CTC-3′, RSP_0799-B: 5′-CAT CAG CTG GTA GCT CTC-3′) as described previously [26].

Oligonucleotides used for gene amplification are listed in Table S5. Conditions for real-time RT-PCR were described earlier in detail [26]. A final concentration of 4 ng µl^−1^ of total RNA was used in an one-step RT-PCR kit (Qiagen). For detection of double stranded DNA SYBR Green I (Invitrogen) was added in a final dilution of 1∶50 000 to the master mix. For normalization of mRNA levels the *rpo*Z gene was used, which encodes the ω-subunit of RNA-polymerase of *R. sphaeroides* [48]. Relative expression of target genes was calculated relative to the expression of untreated samples and relative to *rpoZ* [49]. PCR efficiencies were determined experimentally using serial dilutions of RNA between a final concentration of 8 and 0.5 ng µl^−1^ (Table S6).

### Microarray Analysis

Microarray experiments were performed as described previously [12]. In brief, total RNA from iron-limited and control cultures grown under microaerobic conditions (OD_660_ 0.4) was extracted by the hot phenol method as described earlier [50], [51]. Genomic DNA contamination from RNA samples was removed by DNase treatment (Invitrogen). After DNA digestion, RNA was purified on RNeasy® MinElute™ spin columns (Qiagen). All RNA preparations were tested for the lack of genomic DNA contamination by PCR amplification using primers targeting *gloB* (RSP_0799) as described previously [26].

High-density oligonucleotide *R. sphaeroides* microarrays (Agilent gene chips corresponding to the whole 4.6-Mb genome) were used for transcriptome profiling. The microarray contains probes against 4.304 protein coding genes, 79 rRNA and tRNA genes, and 144 intergenic regions; its construction and performance analysis was performed according to the instructions of Agilent (www.chem.agilent.com). Three antisense probes with a length of 60 nt were designed for hybridization to each gene. The ULS™ Fluorescent Labeling Kit for Agilent arrays (Kreatech) was used for RNA labeling and fragmentation. The RNA of three independent experiments of *R. sphaeroides* wild type under normal and iron limitation conditions and the *irr* deletion mutant under normal and iron limitation conditions was pooled and hybridized to one array. Transcriptome profiles were analyzed on two arrays (Δ*irr* normal iron vs. wild type normal iron; Δ*irr* iron limitation vs. wild type iron limitation; Δ*irr* iron limitation vs. Δ*irr* normal iron) including six biological replicates. Genechip hybridizations and scanning were performed according to the specifications from Agilent. Multiarray analysis was performed with the Bioconductor package Limma for R [52], [53]. Background correction and normalization (Lowess, locally weighted scatterplot smoothing) were performed as described previously [54], [55]. To filter out unreliably measured and unchanged genes, two criteria were used as described previously [12]. (i) Genes were considered reliable if the mean intensity (*A* value) was ≥12. (ii) A cut-off value was applied, i. e., those genes were retained whose average expression value (ratio) was either ≥1.75 or ≤0.57. The fold changes are shown in the text in parentheses preceded by the RSP numbers of their corresponding genes. When expression of several genes is discussed, the lower and upper fold changes are shown, e. g., a range of two- to fivefold increase is shown as “2.0–5.0”. The expression data obtained here were deposited in the Gene Expression Omnibus (GEO) database of the National Center for Biotechnology Information (www.ncbi.nih.gov/geo) under superseries GSE33535.

### Expression and Isolation of the *R. Sphaeroides* Irr Protein

Oligonucleotides 3179-His_fwd (5′-GCGCCCGCAATGGGATGGATCCCATTTC-3′) and 3179-His_rev (5′-GCGGGAATAAGCTTTCAGGTACGCTT-3′) were used for amplifying the coding region of *irr*. The 474-bp PCR product was ligated into the pJET1.2/blunt cloning vector (Qiagen) which was transformed into *E. coli* JM109. Afterwards the plasmid containing *irr* was digested with BamHI and HindIII and the purified *irr* fragment was ligated into the overexpression vector pQE30 (Qiagen) to generate pQE2.4.1*irr*, which was transformed into *E. coli* JM109. The correct construct was transformed into *E. coli* M15 (pREP-4) for overexpression of His-tagged Irr. For this purpose M15 (pREP-4/pQE2.4.1*irr*) was grown in 50 ml of Luria-Bertani medium to an OD_600_ of 0.5 to 0.6 at 37°C. The cells were induced with 1 mM IPTG for 3 h at 37°C. Following harvest, cells were resuspended in ice-cold lysis buffer (50 mM NaH_2_PO_4_, 300 mM NaCl, 10 mM imidazole, pH 8.0) and disrupted by brief sonication. The lysate was centrifuged at 13 000 rpm and 4°C for 15 min. The clear supernatant was loaded onto Ni-NTA agarose (Qiagen) and incubated at 4°C for 3 h. Proteins were washed with washing buffer (50 mM NaH_2_PO_4_, 300 mM NaCl, 20 mM imidazole, pH 8.0) and eluted with elution buffer (50 mM NaH_2_PO_4_, 300 mM NaCl, 250 mM imidazole, pH 8.0). Aliquots of the fractions were analyzed on 15% sodium dodecyl sulfate-polyacrylamide gels, and fractions containing Irr protein were used for the experiments described below.

### Electrophoretic Mobility Shift Assays (EMSAs)

Binding of the recombinant Irr protein to the upstream regions of RSP_0850 (*mbfA*) and RSP_2395 (*ccpA*) was determined by an EMSA. As controls served DNA fragments containing the *sitA* (RSP_0904), the *katE* (RSP_2779) and the *iscR* (RSP_0443) promoter region, respectively. The following oligonucleotides were used to generate DNA fragments containing the respective promoter region by PCR. RSP_0850∶0850up_fwd (5′-GTC AAC TTG CCG CAG GCG CTC C-3′) and 0850up_rev (5′-GCC GGT TGA CAT AGG AGC GGT AG-3′); RSP_2395∶2395up_fwd (5′-CGG TCA ACC CTG GTC GCC GCC GAA-3′) and 2395up_rev (5′-GCC GCG TCG ACG AGG GCC GTC-3′); RSP_0904∶0904up_fwd (5′-CAG TTA ACT GCG AAC GGC TCG CAG A-3′) and 0904up_rev (5′-GAC CGT TAA CGT CGT GGC GAC CT-3′); RSP_0443∶0443up_fwd (5′-CGC GGC GTA ATG TTG ACA AAA ACG-3′) and 0443up_rev (5′-CGA CAC GTC GAC AAG CGA GAC AAG-3′). The PCR fragments with a length of 180, 168, 180 and 246 bp for *mbfA*, *ccpA*, *sitA* and *iscR*, respectively, were cloned into pDrive cloning vector (Qiagen), and isolated from the vector by using the restriction enzyme HincII. In the case of *katE* the plasmid p*katE*up was used that contains a 352 bp fragment of the upstream region of *katE* [29]. The fragment was isolated from the pDrive cloning vector by using the restriction enzymes BamHI and PstI. The restricted DNA fragments were then radioactively end-labeled with γ^32^P ATP using the T4 polynucleotide kinase (Fermentas).

An appropriate amount of the purified Irr protein, ranging from 0.1 to 0.6 µg, was mixed with approx. 3 fmol γ^32^P ATP-labeled DNA probe (5000 c.p.m.) in a 15 µl reaction volume containing 20 mM TB (pH 7.8), 5% v/v glycerol, 1 mM DTT, salmon sperm DNA (1 µg), and 0.1 mg/ml BSA. Binding incubations were carried out for 30 min at 32°C before the samples were loaded onto a 6% polyacrylamide gel in 0.5x TBE buffer (45 mM Tris-HCl, 45 mM boric acid, 1.25 mM EDTA, pH 8.3) and run at 180 V for 3 h at 4°C.

Competitive assays were performed to determine the specificity of the protein for the putative target site (Irr-box). In this case, the γ^32^P ATP-labeled DNA probes were mixed with a 1 to 100 fold molar excess of the respective unlabeled DNA fragment before adding to the binding reaction.

### 5′ RACE

For the determination of 5′ mRNA ends using 5′ rapid amplification of cDNA ends (RACE), 3 µg of total RNA isolated from wild type cells cultivated under normal iron conditions were reverse transcribed into cDNA by using avian myeloblastosis virus reverse transcriptase (Promega) and gene-specific primers (0850_RACE1 and 2395_RACE1; see Table S5). The 5′RACE protocol was performed as described previously [56].

### Heme-binding Experiments

The interaction of Irr with heme was studied through the spectral properties of heme. Hemin (C_34_H_32_ClFeN_4_O_4_; Sigma) was dissolved in 0.1 M NaOH and binding studies were carried out using an appropriate dilution in buffer (50 mM NaH_2_PO_4_, 300 mM NaCl, pH 8.0). The absorption spectrum of 8 µM heme was recorded in the presence or absence of 8 µM Irr. As positive control the absorption spectrum of 5 µM heme was recorded in the presence and absence of 5 µM BSA. As negative control the absorption spectrum of 8 µM heme was recorded in presence and absence of 8 µM IscR.

### Detection of Protoporphyrin IX in Supernatants of *R. sphaeroides* Cultures

40 ml *R. sphaeroides* cells grown to saturation under iron limiting conditions were harvested by centrifugation (4 600 *g*, 15 min, 4°C) and the porphyrins from the supernatants were extracted with 10 ml ethyl/glacial acetic acid (3∶1, v/v) at 18°C overnight with shaking. Then, the ethyl acetate layer was washed with pure water and concentrated at low temperature in a vacuum system. The absorption spectrum of this extract was recorded between 350 and 650 nm with a Specord 50 spectrophotometer (Analytik Jena).

## Supporting Information

Figure S1 Relative gene expression under iron limitation comparing aerobic and microaerobic conditions. Real-time RT-PCR was used to investigate the relative expression of *hemB* (RSP_2848), *hemH* (RSP_1197), *mbfA* (RSP_0850) and *sufD* (RSP_0434) under iron limitation in *R. sphaeroides* 2.4.1Δ*irr* (light gray bars) and wild type (dark gray bars) under microaerobic conditions (non-striped bars) and aerobic conditions (striped bars). Values were normalized to *rpoZ* and to the respective control treatment under normal iron conditions. The data represent the mean of three independent experiments and error bars indicate standard deviation.
(TIF)

Figure S2 Effect of Irr on the absorption spetrum of heme. (A) Absorption spectrum of 8 µM heme was recorded in the absence (dashed line) and in the presence (continuous line) of 8 µM recombinant Irr. A scan of 8 µM Irr alone (dotted line) is also shown. (B) Absorption spectrum of 5 µM heme was recorded in the absence (dashed line) and in the presence (continuous line) of 5 µM BSA as positive control. A scan of 5 µM BSA alone (dotted line) is also shown. (C) Absorption spectrum of 8 µM heme was recorded in the absence (dashed line) and in the presence (continuous line) of 8 µM recombinant IscR. A scan of 8 µM IscR alone (dotted line) is also shown. Absorption peak wavelengths are indicated.
(TIF)

Figure S3 The abundance of small RNAs under iron limitation in the wild type and the 2.4.1Δ*irr* mutant as determined by Northern Blot analysis. After hot phenol extraction RNA was separated on 10% polyacrylamide gels containing 7 M urea and then transferred onto nylon membranes by semidry electroblotting. 10 µg total RNA was loaded per sample. For detection of sRNAs radioactively-labeled oligodeoxynucleotides were used. Membranes were exposed on phosphoimaging screens and analyzed with the 1D-Quantity One software (Bio-Rad). 5 S rRNA served as loading control.
(TIF)

Figure S4 Determination of Irr affinity for Irr-box motif containing DNA. (A) Binding of Irr to the promoter region of *mbfA*. (B) Binding of Irr to the promoter region of *ccpA*. To determine the dissociation constant (K_D_) of Irr-DNA binding, the percentage of DNA bound to total labeled DNA was plotted against increasing Irr concentrations. The K_D_ was defined as the protein concentration required to shift 50% of the probe.
(TIF)

Figure S5 Binding of purified Irr to the promoter region of *katE* and *iscR* as determined by Electrophoretic Mobility Shift Assays. All reactions contain the same amount of ^32^P end-labeled DNA fragment (∼ 3 fmol/lane) comprising the respective promoter sequence. (A) Binding of Irr to the promoter region of *katE* (352 bp). Lanes 1 and 4–6 contain no Irr; lane 6 contains 0.6 µg BSA; lanes 2 and 7 contain 0.1 µg Irr; lane 8 contains 0.2 µg Irr; lane 9 contains 0.3 µg Irr; lane 10 contains 0.4 µg Irr; lanes 11 and 3 contain 0.6 µg Irr. Reactions contain 1 mM MnCl_2_ as indicated. Lanes 1–3 contain radioactively labeled *mbfA* DNA fragment (180 bp) as positive control. (B) Binding of Irr to the promoter region of *iscR* (246 bp). Lanes 1 and 5 contain no Irr; lanes 2 and 6 contain 0.1 µg Irr; lanes 3 and 7 contain 0.3 µg Irr; lanes 4 and 8 contain 0.6 µg Irr. All reactions contain 1 mM MnCl_2_. Lanes 1–4 contain radioactively labeled *mbfA* DNA fragment as positive control. The asterisks and arrows show the location of free and Irr-bound ^32^P end-labeled DNA fragments, respectively.
(TIF)

Figure S6 Confirmation of the *irr* knock-out by PCR (A) using oligodeoxynucleotides KO3179_Test-A (5′-CCA CGC CGA GCG CGA AGC CC-3′) and KO3179_Test-B (5′-GCA CCT CGT CGG GCA GTT CCG-3′) to amplify the *irr* locus with its upstream and downstream regions (estimated product length: WT (− Km^r^ cassette): 1352 bp; Δ*irr* (+ Km^r^ cassette): 2435 bp), (B) using oligodeoxynucleotides KanR2_fwd (5′-CAT GAA CAA TAA AAC TGT CTG C-3′) and KanR2_rev (5′-GAA GAT GCG TGA TCT GAT CC-3′) to amplify the kanamycin resistance cassette (estimated product length: 983 bp) and (C) using oligodeoxynucleotides KanR2_fwd and KO3179_Test-B (estimated product length: Δ*irr* (+ Km^r^ cassette): ∼1800 bp). Used template for PCR: chromosomal DNA (wild type, WT; *irr* deletion mutant, Δ*irr*) and H_2_O as negative control. PCR products were separated on an 1% agarose gel (1x TAE) and stained with ethidium bromide. (D) Construction of *R. sphaeroides* 2.4.1Δ*irr*. Oligodeoxynucleotides used for cloning are indicated as black arrows (A1, A2, B1, B2), oligodeoxynucleotides used for testing knock-out candidates are indicated as red arrows (KO3179_Test-A, KO3179_Test-B) and oligodeoxynucleotides for amplifying the kanamycin resistance cassette are indicated as blue arrows (KanR2_fwd, KanR2_rev).
(TIF)

Table S1 Gene expression changes in 2.4.1Δ*irr*.
(XLS)

Table S2 Selection of iron-responsive genes in *R. sphaeroides* grouped to functional categories.
(DOC)

Table S3 Summary of Irr features in *R. sphaeroides* and Rhizobiales species.
(DOCX)

Table S4 Bacterial strains and plasmids.
(DOC)

Table S5 Oligodeoxynucleotides used for real-time RT-PCR and 5′RACE.
(DOCX)

Table S6 Primer efficiencies for real-time RT-PCR.
(DOCX)
