# Supplementary figures and images for: Role of the Irr Protein in the Regulation of Iron Metabolism in Rhodobacter sphaeroides
Source: PLoS One. 2012 Aug 7;7(8):e42231. doi: 10.1371/journal.pone.0042231 (PMC3413700; doi:10.1371/journal.pone.0042231)

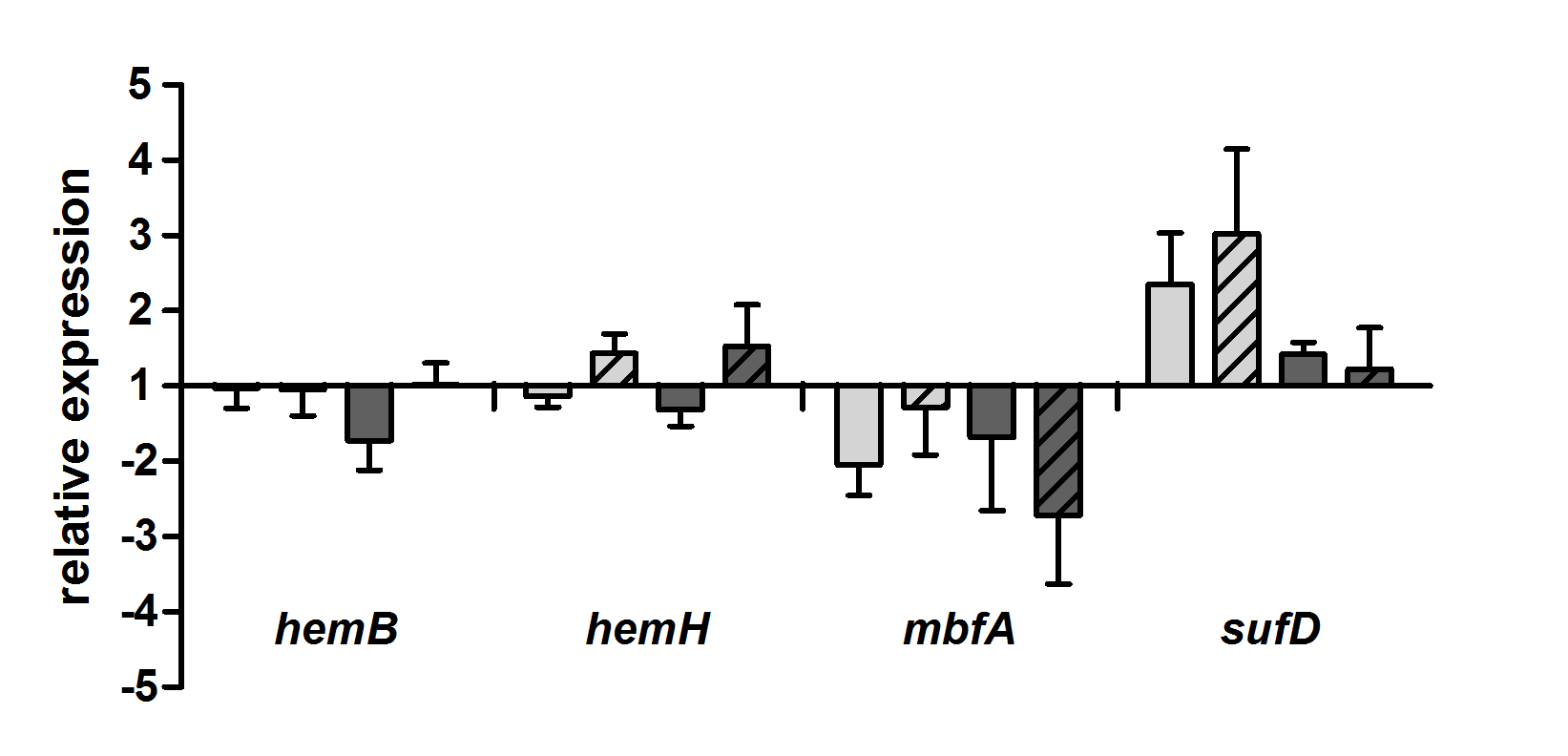

Supplement: Figure S1 — Relative gene expression under iron limitation comparing aerobic and microaerobic conditions. Real-time RT-PCR was used to investigate the relative expression of hemB (RSP_2848), hemH (RSP_1197), mbfA (RSP_0850) and sufD (RSP_0434) under iron limitation in R. sphaeroides 2.4.1Δirr (light gray bars) and wild type (dark gray bars) under microaerobic conditions (non-striped bars) and aerobic conditions (striped bars). Values were normalized to rpoZ and to the respective control treatment under normal iron conditions. The data represent the mean of three independent experiments and error bars indicate standard deviation. (TIF) [file pone.0042231.s001.tif]

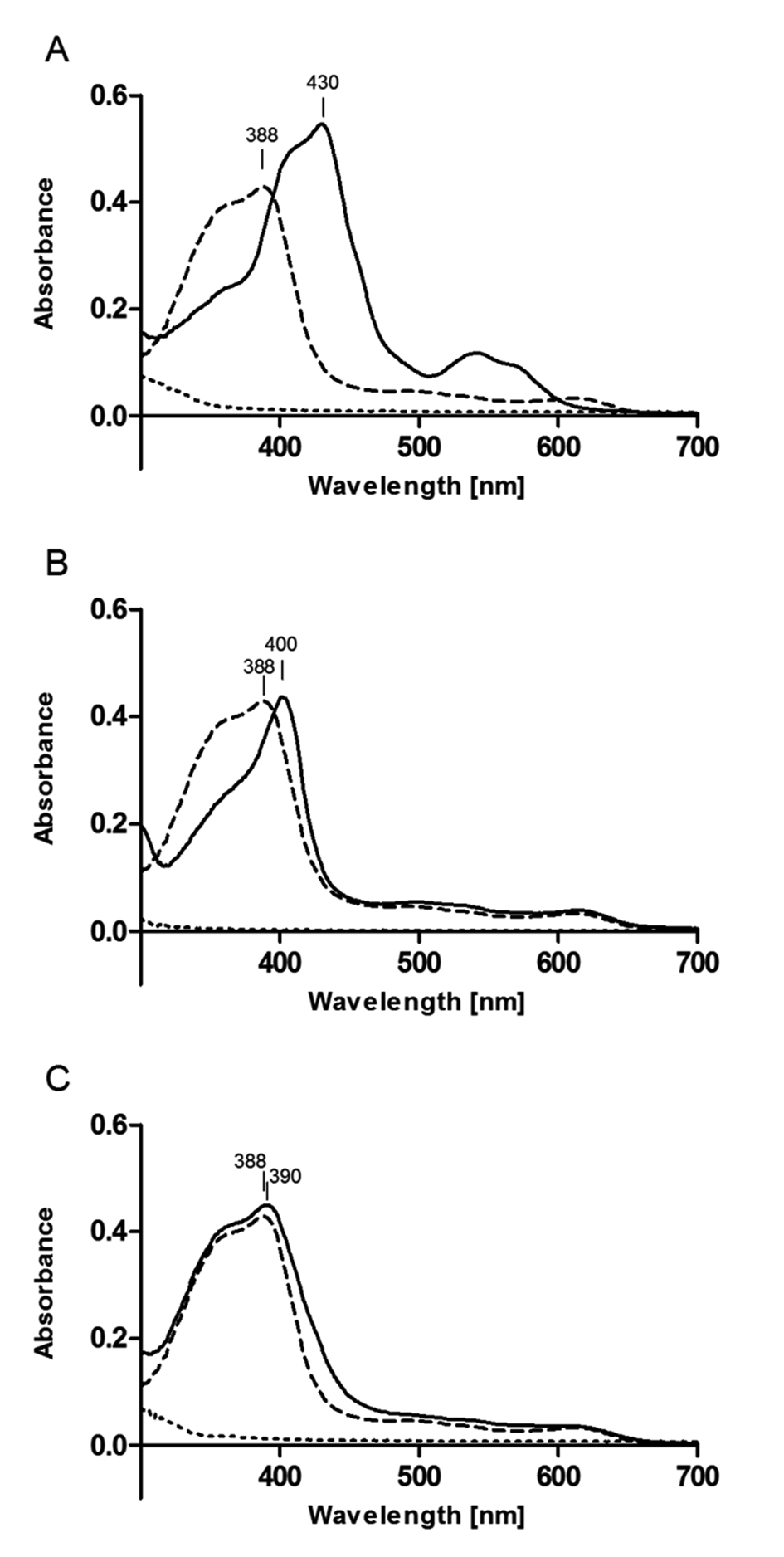

Supplement: Figure S2 — Effect of Irr on the absorption spetrum of heme. (A) Absorption spectrum of 8 µM heme was recorded in the absence (dashed line) and in the presence (continuous line) of 8 µM recombinant Irr. A scan of 8 µM Irr alone (dotted line) is also shown. (B) Absorption spectrum of 5 µM heme was recorded in the absence (dashed line) and in the presence (continuous line) of 5 µM BSA as positive control. A scan of 5 µM BSA alone (dotted line) is also shown. (C) Absorption spectrum of 8 µM heme was recorded in the absence (dashed line) and in the presence (continuous line) of 8 µM recombinant IscR. A scan of 8 µM IscR alone (dotted line) is also shown. Absorption peak wavelengths are indicated. (TIF) [file pone.0042231.s002.tif]

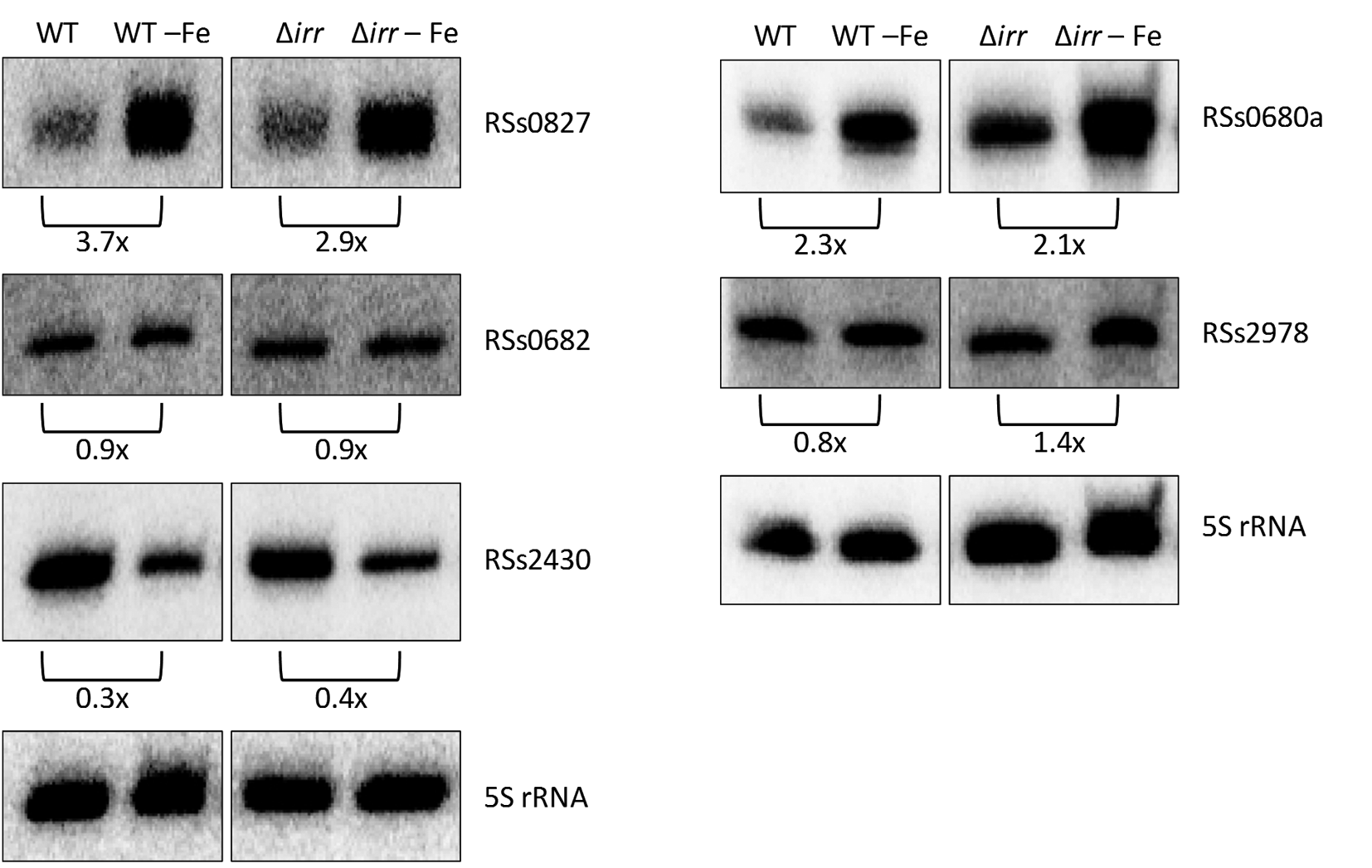

Supplement: Figure S3 — The abundance of small RNAs under iron limitation in the wild type and the 2.4.1Δirr mutant as determined by Northern Blot analysis. After hot phenol extraction RNA was separated on 10% polyacrylamide gels containing 7 M urea and then transferred onto nylon membranes by semidry electroblotting. 10 µg total RNA was loaded per sample. For detection of sRNAs radioactively-labeled oligodeoxynucleotides were used. Membranes were exposed on phosphoimaging screens and analyzed with the 1D-Quantity One software (Bio-Rad). 5 S rRNA served as loading control. (TIF) [file pone.0042231.s003.tif]

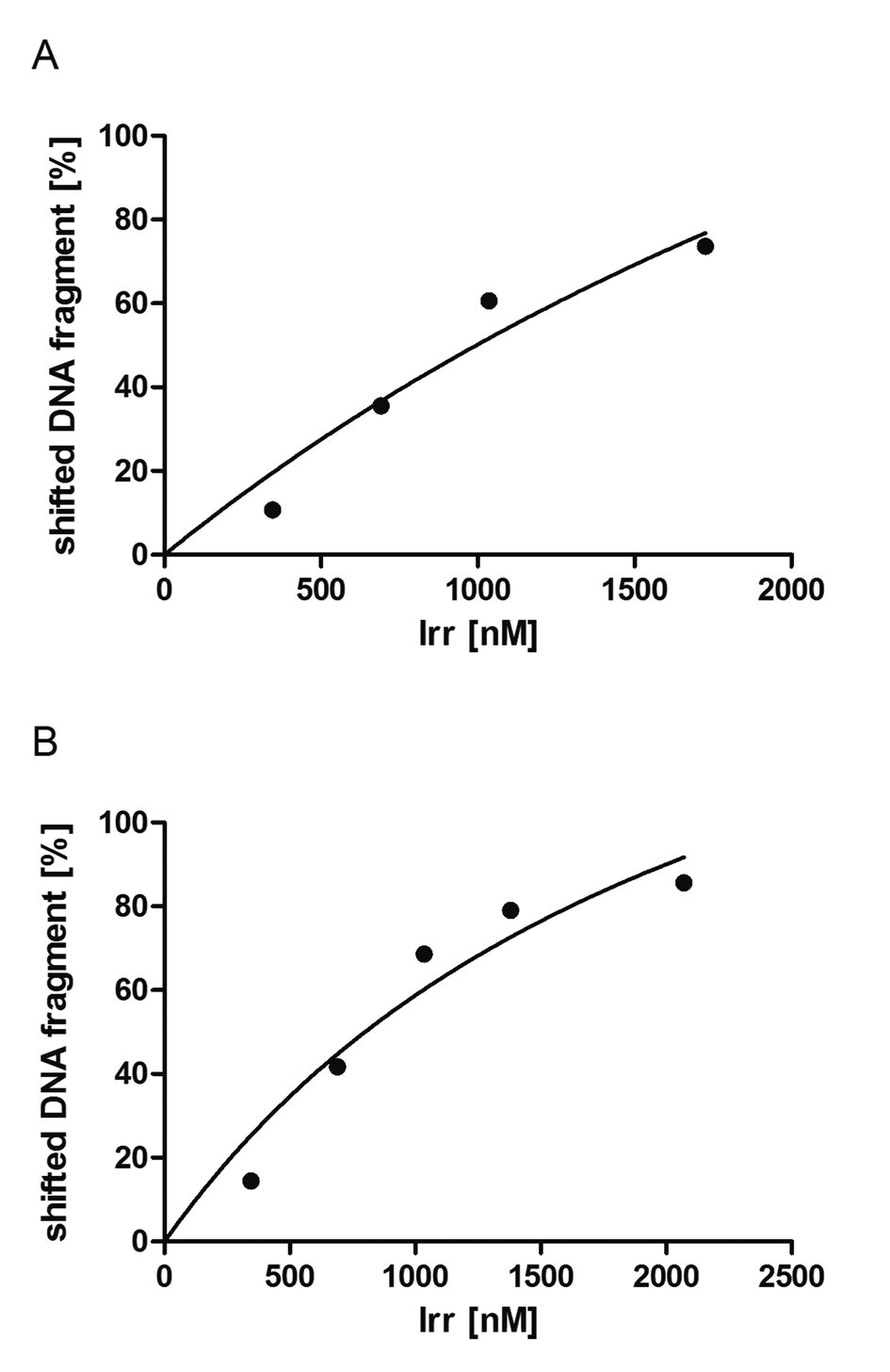

Supplement: Figure S4 — Determination of Irr affinity for Irr-box motif containing DNA. (A) Binding of Irr to the promoter region of mbfA. (B) Binding of Irr to the promoter region of ccpA. To determine the dissociation constant (KD) of Irr-DNA binding, the percentage of DNA bound to total labeled DNA was plotted against increasing Irr concentrations. The KD was defined as the protein concentration required to shift 50% of the probe. (TIF) [file pone.0042231.s004.tif]

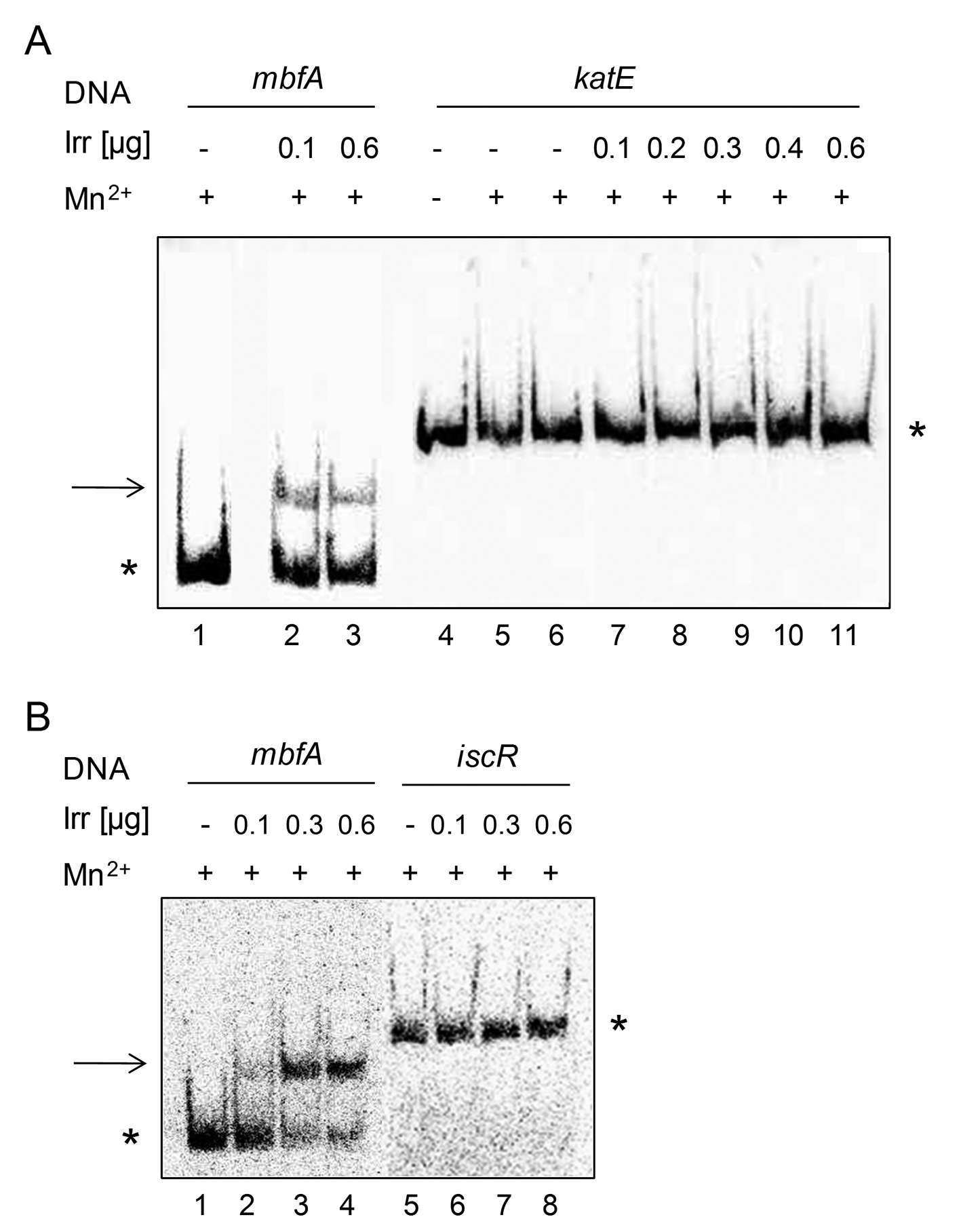

Supplement: Figure S5 — Binding of purified Irr to the promoter region of katE and iscR as determined by Electrophoretic Mobility Shift Assays. All reactions contain the same amount of 32P end-labeled DNA fragment (∼ 3 fmol/lane) comprising the respective promoter sequence. (A) Binding of Irr to the promoter region of katE (352 bp). Lanes 1 and 4–6 contain no Irr; lane 6 contains 0.6 µg BSA; lanes 2 and 7 contain 0.1 µg Irr; lane 8 contains 0.2 µg Irr; lane 9 contains 0.3 µg Irr; lane 10 contains 0.4 µg Irr; lanes 11 and 3 contain 0.6 µg Irr. Reactions contain 1 mM MnCl2 as indicated. Lanes 1–3 contain radioactively labeled mbfA DNA fragment (180 bp) as positive control. (B) Binding of Irr to the promoter region of iscR (246 bp). Lanes 1 and 5 contain no Irr; lanes 2 and 6 contain 0.1 µg Irr; lanes 3 and 7 contain 0.3 µg Irr; lanes 4 and 8 contain 0.6 µg Irr. All reactions contain 1 mM MnCl2. Lanes 1–4 contain radioactively labeled mbfA DNA fragment as positive control. The asterisks and arrows show the location of free and Irr-bound 32P end-labeled DNA fragments, respectively. (TIF) [file pone.0042231.s005.tif]

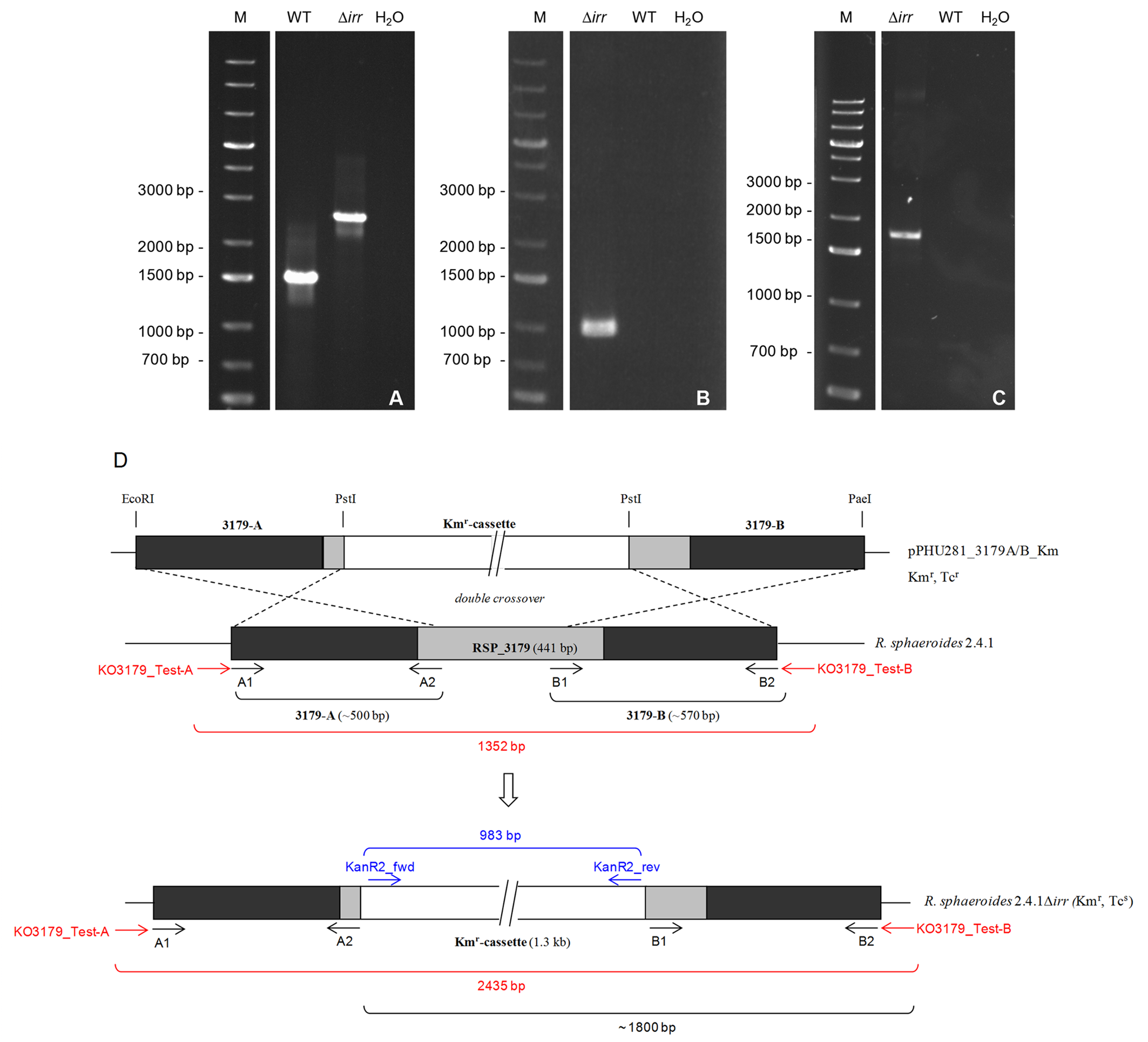

Supplement: Figure S6 — Confirmation of the irr knock-out by PCR (A) using oligodeoxynucleotides KO3179_Test-A (5′-CCA CGC CGA GCG CGA AGC CC-3′) and KO3179_Test-B (5′-GCA CCT CGT CGG GCA GTT CCG-3′) to amplify the irr locus with its upstream and downstream regions (estimated product length: WT (− Kmr cassette): 1352 bp; Δirr (+ Kmr cassette): 2435 bp), (B) using oligodeoxynucleotides KanR2_fwd (5′-CAT GAA CAA TAA AAC TGT CTG C-3′) and KanR2_rev (5′-GAA GAT GCG TGA TCT GAT CC-3′) to amplify the kanamycin resistance cassette (estimated product length: 983 bp) and (C) using oligodeoxynucleotides KanR2_fwd and KO3179_Test-B (estimated product length: Δirr (+ Kmr cassette): ∼1800 bp). Used template for PCR: chromosomal DNA (wild type, WT; irr deletion mutant, Δirr) and H2O as negative control. PCR products were separated on an 1% agarose gel (1x TAE) and stained with ethidium bromide. (D) Construction of R. sphaeroides 2.4.1Δirr. Oligodeoxynucleotides used for cloning are indicated as black arrows (A1, A2, B1, B2), oligodeoxynucleotides used for testing knock-out candidates are indicated as red arrows (KO3179_Test-A, KO3179_Test-B) and oligodeoxynucleotides for amplifying the kanamycin resistance cassette are indicated as blue arrows (KanR2_fwd, KanR2_rev). (TIF) [file pone.0042231.s006.tif]
