# Supplementary material for: Role of the Irr Protein in the Regulation of Iron Metabolism in Rhodobacter sphaeroides
Source: PLoS One. 2012 Aug 7;7(8):e42231. doi: 10.1371/journal.pone.0042231 (PMC3413700; doi:10.1371/journal.pone.0042231)
Supplement: Table S2 — Selection of iron-responsive genes in R. sphaeroides grouped to functional categories. (DOC) [file pone.0042231.s008.doc]

Table S2. Selection of genes that show differential expression in *R. sphaeroides* wild type 2.4.1 and 2.4.1∆*irr* and fall into functional categories

|  | |  | |  |  | |  | | |  |
| --- | --- | --- | --- | --- | --- | --- | --- | --- | --- | --- |
|  | |  | |  | Ratio | | | | |  |
| Category and RSP no. | Gene | | ∆*irr* +Fe  vs.  wild type +Fe *^a^* | | | ∆*irr* -Fe  vs.  wild type -Fe *^a^* | | ∆*irr –*Fe vs.  ∆*irr* +Fe *^a^* | wild type –Fe vs.  wild type +Fe*^b^* | Description |
| **Iron uptake** |  | |  | | |  | |  |  |  |
| RSP_0920 | *exbB* | | (0.66) | | | 1.09 | | **14.82** | **4.29** | Biopolymer transport protein |
| RSP_0921 | *exbD* | | (0.56) | | | 0.92 | | **14.98** | **3.49** | Biopolymer transport protein |
| RSP_0922 |  | | 0.84 | | | 0.99 | | **3.70** | **2.36** | Putative TonB protein |
| RSP_1440 |  | | (0.75) | | | 0.73 | | **7.19** | **3.92** | TonB dependent ferrisiderophore |
| RSP_1548 | *irpA* | | (0.56) | | | 1.25 | | **19.66** | **4.05** | Iron-regulated protein |
| RSP_1818 | *feoB* | | **0.46** | | | 1.01 | | **3.65** | 1.36 | Fe^2+^ transport system protein |
| RSP_1819 | *feoA1* | | **0.49** | | | 1.11 | | **3.72** | 1.55 | Fe^2+^ transport system protein |
| RSP_2913 |  | | (0.46) | | | 0.96 | | **13.21** | **3.22** | ABC Fe^3+^ siderophore transporter |
| RSP_3220 |  | | (0.50) | | | 0.70 | | (7.35) | **1.91** | ABC ferric siderophore transporter |
| RSP_6006 | *hemP* | | (0.64) | | | 1.27 | | **34.65** | **5.18** | Hemin uptake protein |
| RSP_6020 | *feoA2* | | **0.43** | | | 0.86 | | **4.03** | 1.28 | Fe^2+^ transport system protein |
| RSP_7397 |  | | (0.31) | | | 0.81 | | (4.85) | **1.79** | ABC Fe^3+^ siderophore transporter |
| **Iron storage** |  | |  | | |  | |  |  |  |
| RSP_0352 |  | | **0.46** | | | 0.67 | | **2.05** | 1.15 | Probable ferredoxin |
| RSP_0850 | *mbfA* | | **3.65** | | | **8.04** | | **1.75** | (1.47) | Membrane-bound ferritin |
| RSP_1546 | *bfr* | | (0.55) | | | 1.27 | | **4.52** | **1.99** | Bacterioferritin |
| RSP_1547 | *bfd* | | (0.49) | | | 1.32 | | **12.31** | **2.71** | Bacterioferritin-associated ferredoxin |
| RSP_2424 |  | | 0.67 | | | 0.77 | | **1.76** | 1.51 | Ferredoxin II |
| RSP_3342 | *bfr* | | **0.54** | | | 0.70 | | 0.90 | 1.01 | Bacterioferritin |
| **Iron utilization** |  | |  | | |  | |  |  |  |
| RSP_0434 | *sufD* | | 1.48 | | | **2.60** | | **4.32** | **2.46** | Fe-S cluster assembly/repair |
| RSP_0437 | *sufC* | | 1.46 | | | **2.42** | | **4.22** | **1.93** | Fe-S cluster assembly/repair |
| RSP_0439 |  | | 1.50 | | | **2.86** | | **3.42** | **1.81** | Hypothetical protein |
| RSP_0440 | *sufB* | | 1.72 | | | **2.74** | | **3.69** | 1.63 | Fe-S cluster assembly/repair |
| RSP_0442 |  | | (0.74) | | | 1.39 | | (3.92) | 1.56 | Putative aminotransferase |
| RSP_0443 |  | | (0.62) | | | 1.34 | | (4.67) | **1.77** | Rrf2 family transcriptional regulator |
| RSP_2395 | *ccpA* | | 0.90 | | | **1.80** | | **2.32** | 0.78 | BCCP, cytochrome c peroxidase |
| **Stress response** |  | |  | | |  | |  |  |  |
| RSP_0166 | *dksA* | | **0.51** | | | 0.99 | | 1.63 | 1.12 | DnaK suppressor protein |
| RSP_0697 |  | | **0.43** | | | 1.17 | | **1.81** | 0.75 | Universal stress protein |
| RSP_1172 | *dnaJ* | | **0.50** | | | 0.68 | | 1.40 | 1.09 | Chaperone |
| RSP_1194 | *grxC* | | **0.54** | | | 0.96 | | 1.55 | 1.05 | Glutaredoxin |
| RSP_1219 | *grpE* | | **0.57** | | | 0.77 | | 1.74 | 1.11 | Putative chaperone protein GrpE |
| RSP_1529 | *trxA* | | **0.56** | | | 0.82 | | 1.70 | 1.07 | Thioredoxin |
| RSP_1572 |  | | **0.55** | | | 1.47 | | 1.52 | 0.73 | Heat shock protein, Hsp20 family |
| RSP_2310 | *groES* | | 0.72 | | | 0.82 | | **2.31** | 1.49 | Chaperonin Cpn10 (GroES) (protein folding) |
| RSP_2311 | *groEL* | | **0.60** | | | 0.66 | | **2.41** | 1.21 | Chaperonin GroEL |
| RSP_2654 |  | | 0.58 | | | 0.68 | | **1.90** | 1.59 | DnaK suppressor protein |
| RSP_2693 |  | | (0.42) | | | 0.67 | | **2.45** | 1.62 | Superoxide dismutase (Fe-Mn) |
| RSP_2843 | *hfq* | | **0.50** | | | 0.81 | | **1.77** | 1.22 | RNA-binding protein Hfq |
| RSP_4203 |  | | **0.39** | | | 1.03 | | **2.31** | 1.05 | putative glutaredoxin family protein/Thio-disulfide isomerase |
| **Glycolysis / Gluconeo-genesis** |  | |  | | |  | |  |  |  |
| RSP_0404 |  | | **0.56** | | | 0.75 | | **1.80** | 1.43 | Fructose-1,6-bisphosphatase, GlpX-like |
| RSP_1507 |  | | **0.50** | | | 0.74 | | 1.72 | 1.16 | Aldehyde dehydrogenase |
| RSP_1680 | *pckA* | | **0.45** | | | 0.71 | | 1.73 | 1.09 | Phosphoenolpyruvate carboxykinase |
| RSP_2959 | *gapB* | | **0.48** | | | 0.66 | | **2.06** | 1.15 | Glyceraldehyd-3-phosphate dehydrogenase |
| RSP_4047 | *pdhAa* | | **0.52** | | | 0.68 | | **1.90** | 1.28 | Pyruvate dehydrogenase |
| RSP_4049 | *pdhAb* | | **0.48** | | | 0.64 | | **1.82** | 1.26 | Dihydrolipoamide acetyltransferase |
| RSP_4050 | *pdhB* | | **0.47** | | | **0.53** | | **1.82** | 1.03 | Dihydrolipoamide acetyltransferase |
|  |  | |  | | |  | |  |  |  |
| **Citric acid cycle** |  | |  | | |  | |  |  |  |
| RSP_0446 |  | | **0.47** | | | 0.66 | | **1.97** | 1.24 | Isocitrate dehydrogenase |
| RSP_0962 |  | | **0.39** | | | **0.56** | | **1.78** | 1.21 | Dihydrolipoamide drhydrogenase |
| RSP_0964 | *sucB* | | **0.42** | | | **0.51** | | 1.66 | 1.09 | Dihydrolipoamide acetyltransferase |
| RSP_0965 | *sucA* | | **0.46** | | | **0.50** | | **1.82** | 1.59 | 2-oxoglutarate dehydrogenase |
| RSP_0966 | *sucD* | | **0.50** | | | **0.49** | | **1.91** | 1.55 | Succinyl-CoA synthetase |
| RSP_0967 | *sucC* | | **0.48** | | | **0.47** | | **2.06** | **1.79** | Succinyl-CoA synthetase |
| RSP_0968 | *mdh* | | **0.52** | | | 0.65 | | **2.02** | 1.49 | Malate dehydrogenase |
| RSP_0979 | *sdhB* | | **0.49** | | | 0.61 | | 1.54 | 1.35 | Succinate dehydrogenase catalytic subunit |
| RSP_1559 | *icd* | | **0.45** | | | 0.65 | | **2.02** | 1.26 | Isocitrate dehydrogenase |
| RSP_1994 | *gltA* | | **0.46** | | | 0.79 | | **2.04** | 1.21 | Citrate synthase |
| **Oxidative phosphoryla­tion** |  | |  | | |  | |  |  |  |
| RSP_0100 | *nuoA* | | **0.51** | | | 0.97 | | **1.86** | 1.05 | Putative NADH dehydrogenase |
| RSP_0101 | *nuoB* | | **0.54** | | | 1.10 | | **1.83** | 1.04 | NADH dehydrogenase (ubiquinone) |
| RSP_0102 | *nuoCD* | | **0.56** | | | 1.15 | | **1.76** | 0.97 | NADH-ubiquinone oxidoreductase |
| RSP_0103 | *nuoE* | | **0.52** | | | 0.99 | | 1.21 | 0.90 | ATP synthase subunit E |
| RSP_0104 | *nuoF* | | (0.44) | | | 0.83 | | (1.32) | 1.00 | Respiratory-chain NADH dehydrogenase |
| RSP_0105 | *nuoG* | | 0.60 | | | 0.77 | | 1.16 | 1.10 | NADH dehydrogenase |
| RSP_1035 | *atpF* | | **0.49** | | | **0.46** | | **2.11** | 1.62 | F_0_F_1_ ATP synthase |
| RSP_1036 | *atpX* | | **0.50** | | | **0.49** | | **2.24** | **1.75** | F_0_F_1_ ATP synthase |
| RSP_1037 | *atpE* | | **0.45** | | | **0.43** | | **2.41** | 1.58 | ATP synthase |
| RSP_1038 | *atpB* | | **0.51** | | | **0.56** | | **2.12** | 1.68 | ATP synthase |
| RSP_1039 | *atpI* | | **0.54** | | | 0.62 | | **1.89** | 1.23 | F_0_F_1_ ATP synthase |
| RSP_2296 | *atpH* | | **0.56** | | | 0.65 | | **2.05** | 1.50 | ATPase |
| RSP_2297 | *atpA* | | **0.56** | | | 0.66 | | **2.25** | 1.65 | ATP synthase |
| RSP_2298 | *atpG* | | **0.52** | | | 0.60 | | **2.03** | 1.46 | ATP synthase |
| RSP_2299 | *atpD* | | **0.48** | | | **0.56** | | **2.15** | 1.35 | ATP synthase |
| RSP_2300 | *atpC* | | **0.50** | | | 0.63 | | **1.90** | 1.41 | ATP synthase |
| RSP_2512 | *nuoA* | | **0.54** | | | 0.66 | | **2.00** | 1.50 | NADH dehydrogenase |
| RSP_2513 | *nuoB* | | **0.57** | | | 0.72 | | **2.01** | 1.55 | NADH dehydrogenase |
| RSP_2514 | *nuoC* | | **0.53** | | | 0.64 | | **1.97** | 1.56 | NADH dehydrogenase |
| RSP_2515 | *nuoD* | | **0.49** | | | 0.62 | | **2.03** | 1.60 | NADH dehydrogenase |
| RSP_2516 | *nuoE* | | **0.50** | | | 0.58 | | **1.91** | 1.39 | ATP synthase |
| RSP_2518 | *nuoF* | | **0.48** | | | 0.61 | | **1.87** | 1.41 | NADH dehydrogenase-ubiquinone oxidoreductase |
| RSP_2521 | *nuoG* | | (0.46) | | | 0.68 | | **2.07** | 1.36 | NADH dehydrogenase |
| RSP_2522 | *nuoH* | | **0.44** | | | 0.65 | | **2.28** | 1.42 | NADH dehydrogenase |
| RSP_2523 | *nuoI* | | **0.47** | | | 0.70 | | **2.28** | 1.57 | NADH dehydrogenase |
| RSP_2525 | *nuoJ* | | **0.47** | | | 0.71 | | **2.28** | 1.42 | NADH dehydrogenase |
| RSP_2526 | *nuoK* | | **0.46** | | | 0.66 | | **2.20** | 1.30 | NADH dehydrogenase |
| RSP_2527 | *nuoL* | | **0.46** | | | 0.76 | | **2.24** | 1.42 | NADH dehydrogenase |
| RSP_2529 | *nuoM* | | **0.45** | | | 0.69 | | **2.27** | 1.48 | NADH dehydrogenase |
| RSP_2530 | *nuoN* | | **0.45** | | | 0.67 | | **2.35** | 1.45 | NADH dehydrogenase |
| **Fatty acid**  **metabolism** |  | |  | | |  | |  |  |  |
| RSP_0190 | *accB* | | **0.55** | | | 0.70 | | **1.87** | 1.32 | Biotin carboxyl carrier protein |
| RSP_0191 | *accC* | | (0.58) | | | 0.73 | | **1.91** | 1.50 | Acetyl-CoA carboxylase |
| RSP_0745 |  | | **0.53** | | | 0.65 | | 1.64 | 1.23 | Acetyl-CoA acetyltransferase |
| RSP_1256 |  | | **0.32** | | | 0.86 | | **1.90** | 0.75 | Enoyl-(acyl carrier protein) reductase |
| RSP_3833 | *fadB* | | **0.49** | | | 0.73 | | 1.60 | 1.35 | Enoyl-CoA hydratase |
| **Transporter** |  | |  | | |  | |  |  |  |
| RSP_0371 |  | | **0.50** | | | 0.88 | | **1.84** | 1.10 | ABC basic amino acid transporter |
| RSP_0372 |  | | **0.55** | | | 0.75 | | 1.55 | 1.18 | ABC basic amino acid transporter |
| RSP_0910 | *dctP* | | **0.52** | | | **0.41** | | **2.23** | **1.87** | TRAP-T family transporter |
| RSP_0911 | *dctQ* | | 0.62 | | | **0.45** | | **1.95** | **1.90** | TRAP-T family C4-dicarboxylate transporter |
| RSP_0912 | *dctM* | | 0.61 | | | **0.51** | | **1.85** | 1.71 | TRAP-T family C4-dicarboxylate transporter |
| RSP_1747 | *bztA* | | **0.47** | | | 0.83 | | **2.02** | 1.03 | ABC glutamate/glutamine/aspartate /asparagines transporter |
| RSP_1804 | *ccmD* | | **0.53** | | | 1.00 | | 1.72 | 1.08 | Heme exporter protein D |
| RSP_2399 |  | | **0.51** | | | 0.71 | | 1.73 | 1.23 | ABC putrescine transporter |
| RSP_2400 |  | | **0.49** | | | 0.64 | | 1.74 | 1.36 | ABC putrescine transporter |
| RSP_3571 | *znuA* | | (2.98) | | | 0.93 | | (0.26) | (1.62) | ABC zinc transporter |
| **Photo-synthesis** |  | |  | | |  | |  |  |  |
| RSP_0258 | *pufA* | | **0.54** | | | 0.70 | | 1.37 | 0.82 | LHI alpha, Light-harvesting B875 protein |
| RSP_6108 | *pufB* | | **0.57** | | | 0.71 | | 1.28 | 0.68 | LHI beta, light-harvesting B875 subunit |
| RSP_0261 | *bchY* | | 1.34 | | | **2.73** | | **0.52** | 0.82 | Chlorophyllide reductase |
| RSP_0262 | *bchX* | | 1.38 | | | **2.42** | | **0.53** | 0.67 | Chlorophyllide reductase |
| RSP_0263 | *bchC* | | (1.17) | | | **2.35** | | **0.48** | 0.85 | Chlorophyll synthesis pathway |
| RSP_0277 | *bchP* | | 0.95 | | | **1.81** | | 0.88 | 0.95 | Geranylgeranyl hydrogenase |
| RSP_0279 | *bchG* | | 0.71 | | | **1.81** | | 0.95 | 0.65 | bacteriochlorophyll a synthase |
| RSP_0280 | *bchJ* | | **0.55** | | | 1.38 | | **1.89** | 0.76 | Bacteriochlorophyll synthase |
| RSP_0281 | *bchE* | | **0.53** | | | 1.37 | | 1.70 | 0.78 | Magnesium-protoporphyrin IX monomethylester oxidative cyclase |
| RSP_0314 | *pucB* | | 1.30 | | | **3.16** | | **0.51** | **0.57** | LHII beta, light-harvesting B800/850 protein |
| RSP_0315 | *pucC* | | 0.97 | | | **2.57** | | 0.60 | (0.89) | Light-harvesting 1 (B870) complex assembly |
| RSP_0317 | *hemN* | | **0.38** | | | 0.83 | | **2.10** | 0.94 | Coproporphyrinogen III oxidase |
| RSP_6256 | *pucA* | | 1.14 | | | **3.12** | | 0.64 | **0.45** | LHII alpha, light-harvesting B800/850 protein |
| RSP_0679 | *hemC* | | **0.57** | | | 1.07 | | 1.37 | 0.92 | Porphobilinogen deaminase |
| RSP_0680 | *hemE* | | **0.57** | | | 0.93 | | 1.48 | 1.05 | Uroporphyrinogen decarboxylase |
| RSP_0691 | *rdxH* | | **0.49** | | | 0.82 | | **2.08** | 1.15 | RdxH |
| RSP_0692 | *rdxB* | | **0.39** | | | 0.88 | | **2.61** | 1.38 | Iron-sulfur cluster-binding protein |
| RSP_0693 | *ccoP* | | **0.46** | | | 0.65 | | 1.58 | 1.04 | Cbb 3-type cytochrome c oxidase |
| RSP_0694 | *ccoQ* | | **0.47** | | | 0.66 | | 1.53 | 1.03 | Cbb 3-type cytochrome c oxidase |
| RSP_0695 | *ccoO* | | **0.44** | | | 0.63 | | **1.83** | 1.09 | Cbb 3-type cytochrome c oxidase |
| RSP_0696 | *ccoN* | | **0.44** | | | 0.66 | | **2.21** | 1.22 | Cbb 3-type cytochrome c oxidase |
| RSP_0699 | *hemZ* | | **0.56** | | | 0.75 | | **1.84** | 1.64 | Coproporphyrinogen III oxidase |
| RSP_1518 | *prrA* | | **0.57** | | | 0.84 | | 1.66 | 1.07 | Response regulator PrrA |
| RSP_1519 | *prrC* | | **0.48** | | | 0.76 | | **1.90** | 1.19 | PrrC |
| RSP_1556 | *puc2B* | | 1.23 | | | **2.75** | | **0.60** | 0.68 | Light-harvesting complex, beta subunit |
| RSP_6158 | *puc2A* | | 1.08 | | | **2.34** | | 0.70 | **0.56** | Light-harvesting complex, alpha subunit |
| RSP_1565 | *appA* | | **0.47** | | | 0.94 | | **1.92** | 0.93 | AppA, sensor of blue light |
| RSP_1977 | *cobS* | | **0.56** | | | 0.90 | | **1.92** | 1.40 | Cobalt chelatase |
| RSP_2984 | *hemA* | | **0.42** | | | 0.82 | | **2.17** | 0.83 | 5-aminolevulinate synthase |
| **Transcription** |  | |  | | |  | |  |  |  |
| RSP_0386 |  | | **0.47** | | | 0.58 | | 1.47 | 0.90 | Cold-shock DNA-binding domain protein |
| RSP_0395 | *rpoD* | | 0.59 | | | 0.69 | | **1.87** | 1.42 | RNA polymerase sigma factor |
| RSP_0591 |  | | **0.38** | | | **0.43** | | **2.03** | 1.34 | Cold-shock protein |
| RSP_1163 | *nusA* | | **0.52** | | | 1.18 | | **2.28** | 1.02 | Transcription elongation factor |
| RSP_1272 |  | | **0.54** | | | 0.76 | | **2.05** | 1.41 | RNA polymerase sigma-70 factor |
| RSP_1517 | *spbA* | | **0.46** | | | 0.83 | | **1.87** | 1.03 | Histone-like protein of HNS family |
| RSP_1699 | *rpoB* | | **0.56** | | | 0.74 | | **1.87** | 1.26 | DNA-directed RNA polymerase |
| RSP_1704 |  | | 0.63 | | | 0.66 | | **1.94** | 1.69 | Probable transcription antitermination protein NusG |
| RSP_1712 | *rpoC* | | **0.51** | | | 0.64 | | **1.99** | 1.35 | DNA-directed RNA polymerase |
| RSP_1739 | *rpoA* | | 0.61 | | | **0.56** | | **2.09** | **1.89** | DNA-directed RNA polymerase |
| RSP_1952 |  | | **0.42** | | | **0.42** | | **2.04** | 1.22 | Cold-shock DNA-binding domain protein |
| RSP_2024 | *cspA* | | **0.44** | | | **0.45** | | **1.97** | 1.31 | Cold-shock protein |
| RSP_2346 |  | | **0.45** | | | 0.70 | | **2.75** | 1.43 | Cold-shock DNA-binding domain protein |
| RSP_3620 |  | | **0.50** | | | **0.53** | | 1.58 | 0.95 | Cold-shock DNA-binding protein |
| RSP_3621 |  | | **0.42** | | | **0.42** | | **2.11** | 1.19 | Cold-shock DNA-binding protein |
| **RNA processing** |  | |  | | |  | |  |  |  |
| RSP_0819 | *rhlE2* | | 0.59 | | | 0.58 | | **1.84** | 1.62 | DEAD/DEAH box helicase |
| RSP_1060 | *rnpA* | | 0.63 | | | **0.57** | | **1.99** | 1.72 | Ribonuclease P protein component |
| RSP_1112 | *PnP* | | **0.56** | | | **0.51** | | **2.07** | 1.71 | Polyribonucleotide nucleotyltransferase |
| RSP_1971 | *rnd* | | **0.50** | | | 0.77 | | **1.97** | 1.51 | Ribonuclease D |
| RSP_2131 | *rne* | | **0.47** | | | 0.65 | | **1.87** | 1.23 | Ribonuclease E |
| **Amino acid metabolism** |  | |  | | |  | |  |  |  |
| RSP_1109 | *cysK* | | **0.55** | | | 0.72 | | **2.11** | 1.71 | Cysteine synthase |
| RSP_1140 | *ilvE* | | **0.33** | | | **0.54** | | **2.37** | 1.26 | Branched-chain amino acid aminotransferase |
| RSP_2330 | *leuA* | | **0.54** | | | 0.84 | | **1.83** | 1.38 | 2-isopropylmalate synthase |
| RSP_2636 | *ilvH* | | **0.48** | | | 0.69 | | 1.68 | 1.27 | Acetolactate synthase |
| RSP_2637 |  | | **0.41** | | | 0.70 | | **1.84** | 1.05 | Acetolactate synthase |
| RSP_6214 | *trpC* | | **0.48** | | | 0.71 | | 1.73 | 1.12 | Indole-3-glycerol phosphate synthase |
| **Proteolysis** |  | |  | | |  | |  |  |  |
| RSP_0196 | *clpX* | | **0.54** | | | 0.98 | | **1.93** | 1.08 | ATP-dependent protease ATP-binding subunit |
| RSP_0197 | *clpP* | | **0.55** | | | 1.01 | | 1.74 | 1.05 | Protease subunit of ATP-dependent Clp protease |
| RSP_0355 |  | | **0.55** | | | 0.85 | | **1.77** | 1.15 | Possible serine protease |
| RSP_0356 |  | | **0.52** | | | 0.77 | | 1.58 | 1.11 | Probable HflC protein |
| RSP_0357 |  | | **0.52** | | | 0.83 | | 1.66 | 0.96 | Probable HflK protein |
| RSP_0464 |  | | **0.45** | | | 0.82 | | **1.84** | 0.83 | Putative protease |
| RSP_0465 |  | | **0.53** | | | 0.88 | | **1.86** | 1.13 | Putative protease |
| RSP_0665 | *ftsH* | | **0.53** | | | 0.96 | | **1.86** | 1.03 | ATP-dependent Zn metalloprotease |
| **Ribosomal proteins/translation** |  | |  | | |  | |  |  |  |
| RSP_0021 | *rpsI* | | 0.58 | | | 0.72 | | **2.12** | 1.50 | 30S ribosomal protein S9 |
| RSP_0022 |  | | **0.54** | | | 0.66 | | **2.31** | 1.41 | 50S ribosomal protein L13 |
| RSP_0139 |  | | **0.56** | | | **0.44** | | **2.31** | **1.89** | Ribosomal protein S6 |
| RSP_0140 | *rpsR* | | **0.57** | | | **0.47** | | **2.28** | **1.88** | 30S ribosomal protein S18 |
| RSP_0141 |  | | 0.58 | | | **0.44** | | **2.31** | **1.76** | 50S ribosomal protein L9 |
| RSP_0448 |  | | 0.65 | | | 0.62 | | **1.76** | 1.64 | EF-Tu; elongation factor Tu |
| RSP_0626 | *infA* | | **0.56** | | | 0.71 | | **1.99** | 1.35 | Translation initiation factor IF-1 |
| RSP_0718 | *rpsU* | | **0.42** | | | **0.45** | | **2.31** | 1.39 | 30S ribosomal protein S21 |
| RSP_0827 |  | | **0.54** | | | **0.51** | | **2.13** | 1.62 | Ribosomal protein L25 |
| RSP_1043 | *rpmE* | | 0.58 | | | **0.51** | | **2.01** | 1.60 | 50S ribosomal protein L31 |
| RSP_1044 | *rplS* | | 0.58 | | | **0.50** | | **1.97** | **1.97** | 50S ribosomal protein L19 |
| RSP_1048 | *rpsP* | | **0.47** | | | **0.44** | | **1.99** | **1.93** | 30S ribosomal protein S16 |
| RSP_1049 | *pheAa* | | (0.52) | | | **0.56** | | (1.84) | 1.65 | Chorismate mutase |
| RSP_1059 | *rpmH* | | 0.58 | | | **0.56** | | **2.23** | **1.78** | 50S ribosomal protein L34 |
| RSP_1111 | *rpsO* | | **0.50** | | | **0.38** | | **2.11** | 1.42 | Ribosomal protein S15 |
| RSP_1157 | *PSrp1* | | **0.54** | | | 1.02 | | **1.93** | 1.01 | Ribosomal subunit interface protein Y |
| RSP_1165 | *infB* | | **0.53** | | | 0.76 | | 1.72 | 1.10 | Translation initiation factor IF-2 |
| RSP_1341 | *rpsT* | | 0.60 | | | **0.55** | | **2.41** | 1.69 | Ribosomal protein S20 |
| RSP_1398 | *rpmJ* | | 0.61 | | | **0.55** | | **2.02** | **1.96** | Ribosomal protein L36 |
| RSP_1700 | *rplL* | | **0.41** | | | **0.38** | | **2.45** | 1.29 | 50S ribosomal protein L7/L12 |
| RSP_1701 | *rplJ* | | **0.40** | | | **0.36** | | **2.47** | 1.26 | Ribosomal protein L10 |
| RSP_1702 | *rplA* | | **0.52** | | | **0.51** | | **2.15** | 1.49 | 50S ribosomal protein L1 |
| RSP_1703 | *rplK* | | **0.54** | | | **0.53** | | **2.24** | 1.48 | Ribosomal protein L11 |
| RSP_1707 | *tufA* | | **0.50** | | | **0.45** | | **2.36** | 1.40 | Elongation factor Tu (EF-Tu) |
| RSP_1708 | *fusA1* | | 0.60 | | | **0.51** | | **2.20** | 1.73 | Elongation factor G |
| RSP_1709 | *rpsG* | | 0.63 | | | **0.53** | | **2.26** | **1.95** | 30S ribosomal protein S7 |
| RSP_1710 | *rpsL* | | 0.58 | | | **0.44** | | **2.39** | **2.06** | 30S ribosomal protein S12 |
| RSP_1714 | *tufA* | | **0.50** | | | **0.45** | | **2.36** | 1.45 | Elongation factor TU |
| RSP_1715-22 |  | | **0.49-0.50** | | | **0.42-0.51** | | **2.17-2.40** | 1.39-1.63 | Ribosomal proteins |
| RSP_1723 | *rplP* | | **0.42** | | | **0.39** | | **2.45** | 1.22 | 50S ribosomal protein L16 |
| RSP_1724-34 |  | | **0.45**-0.62 | | | **0.40-0.54** | | **1.98-2.31** | 1.33-**2.09** | Ribosomal proteins |
| RSP_1735 | *secY* | | **0.57** | | | 0.67 | | **1.76** | 1.40 | Preprotein translocase SecY |
| RSP_1736 | *adk* | | **0.56** | | | 0.70 | | 1.67 | 1.29 | Adenylate kinase |
| RSP_1737-38 |  | | **0.53-0.56** | | | **0.42-0.45** | | **2.03-2.10** | 1.64-**1.76** | 30S ribosomal proteins |
| RSP_1740 |  | | **0.54** | | | **0.53** | | **2.24** | 1.49 | Ribosomal protein L17 |
| RSP_1764 | *rplT* | | 0.62 | | | **0.57** | | **2.21** | 1.69 | 50S ribosomal protein L20 |
| RSP_1765 | *rpmI* | | 0.65 | | | 0.59 | | **2.23** | **1.91** | 50S ribosomal protein L35 |
| RSP_1887 |  | | 0.61 | | | **0.53** | | **2.29** | **1.78** | 50S ribosomal protein L33 |
| RSP_1937 | *infC* | | **0.50** | | | 0.70 | | **2.00** | 1.45 | Translation initiation factor IF-3 |
| RSP_2016 | *rpmB* | | **0.56** | | | **0.53** | | **2.33** | 1.52 | 50S ribosomal protein L28 |
| RSP_2247 | *fusA* | | **0.36** | | | 1.44 | | **1.96** | 0.67 | translation elongation factor EF-G |
| RSP_2283 | *rpsD* | | **0.57** | | | **0.40** | | **2.41** | **2.08** | 30S ribosomal protein S4 |
| RSP_2614 | *rpmF* | | 0.67 | | | 0.58 | | **2.12** | **1.82** | possible 50S ribosomal protein L32 |
| RSP_2706 | *frr* | | 0.58 | | | 0.73 | | **1.78** | 1.32 | Ribosome recycling factor |
| RSP_2860 | *rpsB* | | **0.56** | | | 0.73 | | **2.35** | 1.49 | 30S ribosomal protein S2 |
| RSP_2861 | *tsf* | | 0.58 | | | 0.67 | | **2.23** | 1.59 | elongation factor Ts |
| RSP_3590 |  | | **0.47** | | | **0.49** | | **2.16** | 1.71 | 30S ribosomal protein S1 |
| RSP_3819 | *rplU* | | **0.57** | | | **0.49** | | **2.32** | (1.65) | Ribosomal protein L21 |
| RSP_3820 | *rpmA* | | 0.64 | | | 0.61 | | **2.11** | 1.33 | 50S ribosomal protein L27 |
| RSP_6017 | *rpsQ* | | 0.58 | | | **0.44** | | **2.15** | **2.12** | Ribosomal protein S17 |
| **Cell division** |  | |  | | |  | |  |  |  |
| RSP_2106 | *ftsW* | | **0.57** | | | 0.83 | | 1.66 | 1.22 | Cell division protein |
| RSP_2112 | *ftsQ* | | **0.55** | | | 0.61 | | 1.74 | 1.52 | Cell division septal protein |
| RSP_2113 | *ftsA* | | **0.53** | | | 0.61 | | **1.80** | 1.48 | Cell division protein |
| RSP_2114 | *ftsZ1* | | **0.55** | | | 0.68 | | **1.78** | 1.15 | Cell division protein |
| **Chemotaxis** |  | |  | | |  | |  |  |  |
| RSP_0037 | *flgM* | | **0.38** | | | **0.48** | | (0.65) | **0.57** | Negative regulator of flagellin synthesis |
| RSP_0039 |  | | **0.42** | | | 0.71 | | 0.98 | 0.76 | hypothetical protein |
| RSP_0040 | *fliS* | | **0.27** | | | **0.55** | | (0.47) | **0.46** | Flagellar protein |
| RSP_0042 | *cheA3* | | (0.24) | | | **0.52** | | (0.60) | 0.66 | Chemotaxis histidine protein kinase |
| RSP_0043 | *cheY6* | | (0.21) | | | **0.46** | | (0.52) | **0.57** | Chemotaxis response regulator |
| RSP_0046 | *cheW4* | | (0.25) | | | 0.63 | | (0.61) | 0.85 | Chemotaxis protein |
| RSP_0049 | *cheA4* | | **0.34** | | | 0.80 | | (0.53) | 0.61 | Chemotaxis histidine protein kinase |
| RSP_0069 | *fliC* | | **0.19** | | | **0.55** | | (0.50) | **0.30** | Flagellar filament protein |
| RSP_0080 | *flgE* | | (0.31) | | | 0.78 | | (1.65) | 1.16 | flagellar hook protein FlgE |
| RSP_0082 | *flgC* | | (0.32) | | | 0.64 | | (1.42) | 1.09 | Flagellar basal-body rod protein |
| RSP_0083 | *flgB* | | (0.29) | | | 0.70 | | (1.69) | 0.95 | Flagellar proximal rod protein FlgB |
| RSP_0149 |  | | 0.52 | | | 0.79 | | **1.92** | 1.44 | CheY-like receiver protein |
| RSP_1583 | *cheY3* | | (0.27) | | | 0.84 | | (1.10) | 0.69 | Chemotaxis response regulator |
| RSP_1585 | *cheW2* | | **0.44** | | | 0.69 | | (0.88) | 0.85 | Chemotaxis protein |
| **Other categories** |  | |  | | |  | |  |  |  |
| RSP_0252 |  | | **0.47** | | | 0.68 | | **2.00** | 1.21 | Protein-L-isoaspartate carboxylmethyltransferase |
| RSP_0820 |  | | **0.45** | | | 1.10 | | **1.78** | 0.84 | Putative cytochrome B561 (respiratory electron transport chain) |
| RSP_0842 |  | | **0.28** | | | **0.30** | | **2.82** | 0.97 | Putative porin |
| RSP_0843 |  | | **0.43** | | | 0.58 | | **1.80** | 1.16 | Conserved protein containing sulfotransfer domain |
| RSP_1468 |  | | **0.47** | | | 0.60 | | 1.68 | 1.35 | Site-specific DNA-methyltransferase |
| RSP_2536 | *rhlE* | | **0.53** | | | 0.62 | | **1.97** | 1.33 | ATP-dependent helicase. DEAD-box |
| RSP_2888 |  | | **0.49** | | | 1.14 | | **1.87** | 0.92 | Transcriptional regulator |
| RSP_4242 |  | | **0.41** | | | 0.78 | | 1.49 | 0.92 | Hemolysin-type calcium-binding protein |
| RSP_6096 |  | | **0.49** | | | 0.79 | | **1.95** | 1.49 | Transcriptional regulator. LuxR family |

*^a^* Significant changes are in bold. Numbers in parentheses failed to meet the set *A* value criteria, while plain numbers show a lower fold change than ≥ 1.75 or ≤ 0.57. Selected genes that missed the cut-offs are included in this table to fully represent functional groups discussed.

*^b^* Values are taken from Peuser and colleagues (2011).
