# Supplementary material for: Role of the Irr Protein in the Regulation of Iron Metabolism in Rhodobacter sphaeroides
Source: PLoS One. 2012 Aug 7;7(8):e42231. doi: 10.1371/journal.pone.0042231 (PMC3413700; doi:10.1371/journal.pone.0042231)
Supplement: Table S3 — Summary of Irr features in R. sphaeroides and Rhizobiales species. (DOCX) [file pone.0042231.s009.docx]

Table S3. Summary of Irr features in *R. sphaeroides* and Rhizobiales species

| Feature | *Rhodobacter sphaeroides* | *Brucella abortus*^1,2^ | *Rhizobium leguminosarum*^3,4,5^ | *Bradyrhizobium japonicum*^6,7^ |
| --- | --- | --- | --- | --- |
| Heme Regulatory Motif (HRM) | no | no | no | yes |
| Heme Binding Motif (HXH) | HPH | HQH | HHH | HHH |
| Heme Binding | yes | yes | yes | yes |
| Protoporphyrin accumulation in ∆*irr* -Fe | no | yes | yes | yes |
| Growth behavior of ∆*irr* -Fe | Similar like wild type | Similar like wild type | * | * |
| ∆*irr*: resistance to oxidative stress | Higher resistance | Higher resistance | * | * |
| Irr represses ALA dehydratase expression (*hemB*) | no | * | yes | yes |
| *irr* transcription under Fe limitation | unchanged | unchanged | induced | induced |
| Irr box | *mbfA*, *ccpA* | *mbfA*? | *mbfA*, *irpA*, *fumA*, *hemA1* | *hmuRSTUV*, *exbBD-tonB*, *feoAB*, *mbfA*, *bfd-bfr*, *suf*, *katG*, *fumA*, *ccm* |
| RirA (Rhizobial iron regulator) | no | yes | yes | yes |
| IscR (Iron sulfur cluster regulator) | yes | no | no | no |

* no information available

^1^ Martinez M, Ugalde RA, Almiron M (2005) Dimeric *Brucella abortus* Irr protein controls its own expression and binds haem. Microbiology 151: 3427-3433.

^2^ Martinez M, Ugalde RA, Almiron M (2006) Irr regulates brucebactin and 2,3-dihydroxybenzoic acid biosynthesis, and is implicated in the oxidative stress resistance and intracellular survival of *Brucella abortus*. Microbiology 152: 2591-2598.

^3^ Wexler M, Todd JD, Kolade O, Bellini D, Hemmings AM, et al. (2003) Fur is not the global regulator of iron uptake genes in *Rhizobium leguminosarum*. Microbiology 149: 1357-1365.

^4^ Todd JD, Sawers G, Rodionov DA, Johnston AW (2006) The *Rhizobium leguminosarum* regulator IrrA affects the transcription of a wide range of genes in response to Fe availability. Mol Genet Genomics 275: 564-577.

^5^ Singleton C, White GF, Todd JD, Marritt SJ, Cheesman MR, et al. Heme-responsive DNA binding by the global iron regulator Irr from *Rhizobium leguminosarum*. J Biol Chem 285: 16023-16031.

^6^ Hamza I, Chauhan S, Hassett R, O'Brian MR (1998) The bacterial *irr* protein is required for coordination of heme biosynthesis with iron availability. J Biol Chem 273: 21669-21674.

^7^ Rudolph G, Semini G, Hauser F, Lindemann A, Friberg M, et al. (2006) The Iron control element, acting in positive and negative control of iron-regulated *Bradyrhizobium japonicum* genes, is a target for the Irr protein. J Bacteriol 188: 733-744.
