# Supplementary material for: Role of the Irr Protein in the Regulation of Iron Metabolism in Rhodobacter sphaeroides
Source: PLoS One. 2012 Aug 7;7(8):e42231. doi: 10.1371/journal.pone.0042231 (PMC3413700; doi:10.1371/journal.pone.0042231)
Supplement: Table S4 — Bacterial strains and plasmids. (DOC) [file pone.0042231.s010.doc]

**Table S4.** Bacterial strains and plasmids

| Strain or plasmid | Description* | Reference or source |
| --- | --- | --- |
| ***E. coli* strains** |  |  |
| JM109 | Cloning strain | New England Biolabs |
| S17-1 | Donor strain for diparental conjugation | Simon et al., 1986 ^1^ |
| M15 (pREP-4) | Host strain for protein overexpression | Qiagen |
| M15 (pREP-4/pQE2.4.1*irr*) | Irr overexpression strain | this study |
|  |  |  |
| ***R. sphaeroides* strains** |  |  |
| 2.4.1 | Wild-type | van Niel, 1944 ^2^ |
| 2.4.1∆*irr* | 2.4.1∆*irr*::Km | this study |
| 2.4.1∆*irr* (pRK2.4.1*irr*) | 2.4.1∆*irr* harbouring pRK2.4.1*irr* | this study |
|  |  |  |
| **Plasmids** |  |  |
| pPHU281 | Tc^r^, *lacZ’ mob*(RP4) | Hübner et al., 1991 ^3^ |
| pUC4K | source of Km^r^ cassette | Vieira & Messing, 1982 ^4^ |
| pQE30 | Ap^r^, 3.4 kb, 6xHis-tag overexpression vector | Qiagen |
| pDrive cloning vector | Ap^r^, Km^r^, 3.85 kb | Qiagen |
| pJET1.2/blunt cloning vector | Ap^r^, 2.97 kb | Fermentas |
| pPHU∆2.4.1*irr* | 1.08 kb fragment containing the upstream and downstream regions of the *R. sphaeroides irr* gene (RSP_3179) cloned into pPHU281 | this study |
| pPHU∆2.4.1*irr*::Km | 1.3 kb kanamycin cassette cloned into pPHU∆2.4.1*irr* | this study |
| pRK2.4.1*irr* | 539 bp fragment containing the *irr* gene and its upstream and downstream regions cloned into pRK415 | this study |
| pQE2.4.1*irr* | 474 bp BamHI/HindIII DNA fragment containing the 2.4.1_*irr* gene cloned into pQE30 for Irr overexpression | this study |
| p*mbfA*up | 180 bp HincII DNA fragment containing the upstream region of *mbfA* (RSP_0850) cloned into pDrive cloning vector | this study |
| p*ccpA*up | 168 bp HincII DNA fragment containing the upstream region of *ccpA* (RSP_2395) cloned into pDrive cloning vector | this study |
| p*sitA*up | 180 bp HincII DNA fragment containing the upstream region of *sitA* (RSP_0904) cloned into pDrive cloning vector | this study |
| p*katE*up | 352 bp BamHI and PstI DNA fragment containing the upstream region of 2.4.1 *katE* (RSP_2779) cloned into pDrive cloning vector | Zeller & Klug, 2004 ^5^ |

* Tc^r^, tetracycline-resistant; Ap^r^, ampicillin-resistant; Km^r^, kanamycin-resistant

^1^ Simon, R., O'Connell, M., Labes, M. & Pühler, A. (1986) Plasmid vectors for the genetic analysis and manipulation of Rhizobia and other gram-negative bacteria. Methods Enzymol 118: 640-659.

^2^ van Niel, C. B. (1944) The Culture, General Physiology, Morphology, and Classification of the Non-Sulfur Purple and Brown Bacteria. Bacteriol Rev 8: 1-118.

^3^ Hübner, P., Willison, J. C., Vignais, P. M. & Bickle, T. A. (1991) Expression of regulatory *nif* genes in *Rhodobacter capsulatus*. J Bacteriol 173: 2993-2999.

^4^ Vieira, J. & Messing, J. (1982) The pUC plasmids, an M13mp7-derived system for insertion mutagenesis and sequencing with synthetic universal primers. Gene 19: 259-268.

^5^ Zeller, T. & Klug, G. (2004). Detoxification of hydrogen peroxide and expression of catalase genes in *Rhodobacter*. Microbiology 150: 3451-3462.
