# Supplementary material for: Role of the Irr Protein in the Regulation of Iron Metabolism in Rhodobacter sphaeroides
Source: PLoS One. 2012 Aug 7;7(8):e42231. doi: 10.1371/journal.pone.0042231 (PMC3413700; doi:10.1371/journal.pone.0042231)
Supplement: Table S5 — Oligodeoxynucleotides used for real-time RT-PCR and 5′RACE. (DOCX) [file pone.0042231.s011.docx]

**Table S5.** Oligodeoxynucleotides used for real-time RT-PCR and 5’RACE.

| **Oligodeoxynucleotide** | **Sequence** | **Reference** |
| --- | --- | --- |
| rpoZ-A | 5’-ATCGCGGAAGAGACCCAGAG-3’ | Gomelsky et al.^1^ |
| rpoZ-B | 5’-GAGCAGCGCCATCTGATCCT-3’ | Gomelsky et al.^1^ |
| RSP1547RT-A | 5’-GTCTGCCACTGCATGGGCAT-3’ | Peuser et al.^2^ |
| RSP1547RT-B | 5’-GTTCATTCCTGCGGTTGTGG-3’ | Peuser et al.^2^ |
| RSP0434_RT-A | 5’-CGCGGTTCTGCACCATTG-3’ | this study |
| RSP0434_RT-B | 5’-TTGGCCGTCAGCGTGAAG-3’ | this study |
| 2395_RT_A | 5’-TCGAGGCGACGCTCATCA-3’ | this study |
| 2395_RT_B | 5’-TCGATCAGGCCGAACGGA-3’ | this study |
| RSP0440_sufB-A | 5’-ACATCGTGCGGCTGATCT-3’ | this study |
| RSP0440_sufB-B | 5’-CGGCTTCACATCCATGCT-3’ | this study |
| RSP2779RT-A | 5’-CTATCCGCTGATCGAGGT-3’ | Zeller et al.^3^ |
| RSP2779RT-B | 5’-GTCGGCATAGGAGAAGAC-3’ | Zeller et al.^3^ |
| 3571_RT-A | 5’-GATCACGGCGAACATGAG-3’ | this study |
| 3571_RT-B | 5’-GGCATCGTGGAAGACGTA-3’ | this study |
| RSP0850_RT-A | 5’-GCGTCGGCAGTTCATCCT-3’ | this study |
| RSP0850_RT-B | 5’-GAGAGCACGCCATCGTCA-3’ | this study |
| 0922_up | 5’-TCCGAGACCTTCGCCTAC-3’ | this study |
| 0922_down | 5’-GCCGTCAGGTTCTGCACT-3 | this study |
| RSP_1197_RT-A | 5’-GCCGGACAATTACGACTA-3‘ | this study |
| RSP_1197_RT-B | 5’-TGTCTGGTCCTTGGTGAT-3’ | this study |
| RSP_2848_RT-A | 5’-ATGTGGCGATCCTCTCCT-3‘ | this study |
| RSP_2848_RT-B | 5’-CCGGCAGATGTCGAGATA-3’ | this study |
| 0850_RACE1 | 5’-GCAGCGTTCTCCATCTCCCAAG-3’ | this study |
| 0850_RACE2 | 5’-GCGGGATCGTCTCGCCGAACC-3’ | this study |
| 2395_RACE1 | 5’-CGTTCAGCACCGCATTGAGCAC-3’ | this study |
| 2395_RACE2 | 5‘-GAATAGCAGGAGAAGACGCCCG-3‘ | this study |
| Oligo d(T)-anchor primer | 5’-GACCACGCGTATCGATGTCGACTTTTTTT-  TTTTTTTTTV-3’; V=A, C or G | Roche |
| PCR anchor primer | 5’-GACCACGCGTATCGATGTCGAC-3’ | Roche |
|  |  |  |

^1^ Gomelsky, L., Sram, J., Moskvin, O. V., Horne, I. M., Dodd, H. N., Pemberton, J. M. et al. (2003) Identification and in vivo characterization of PpaA, a regulator of photosystem formation in *Rhodobacter sphaeroides*. Microbiology 149: 377-388.

^2^ Peuser, V., Metz, S. & Klug, G. (2011) Response of the photosynthetic bacterium *Rhodobacter sphaeroides* to iron limitation and the role of a Fur orthologue in this response. Environ Microbiol Rep 3: 397-404.

^3^ Zeller, T. & Klug, G. (2004) Detoxification of hydrogen peroxide and expression of catalase genes in *Rhodobacter*. Microbiology 150: 3451-3462.
