# Supplementary material for: Role of the Irr Protein in the Regulation of Iron Metabolism in Rhodobacter sphaeroides
Source: PLoS One. 2012 Aug 7;7(8):e42231. doi: 10.1371/journal.pone.0042231 (PMC3413700; doi:10.1371/journal.pone.0042231)
Supplement: Table S6 — Primer efficiencies for real-time RT-PCR. (DOCX) [file pone.0042231.s012.docx]

**Table S6.** Primer efficiencies for real-time RT-PCR.

| **Corresponding gene** | **Efficiency** | **Reference** |
| --- | --- | --- |
| RSP_0434 (*sufD*) | 1.97 | this study |
| RSP_0440 (*sufB*) | 2.11 | this study |
| RSP_0850 (*mbfA*) | 2.09 | this study |
| RSP_1547 (*bfd*) | 1.81 | Peuser et al.^1^ |
| RSP_1669 (*rpoZ*) | 2.02 | Gomelsky et al.^2^ |
| RSP_2395 (*ccpA*) | 2.08 | this study |
| RSP_2779 (*katE*) | 2.07 | Zeller et al.^3^ |
| RSP_3571 (*znuA*) | 2.03 | this study |
| RSP_0922 (*tonB*) | 1.77 | this study |
| RSP_1197 (*hemH*) | 1.76 | this study |
| RSP_2848 (*hemB*) | 1.77 | this study |

^1^ Peuser, V., Metz, S. & Klug, G. (2011) Response of the photosynthetic bacterium *Rhodobacter sphaeroides* to iron limitation and the role of a Fur orthologue in this response. Environ Microbiol Rep 3: 397-404.

^2^ Gomelsky, L., Sram, J., Moskvin, O. V., Horne, I. M., Dodd, H. N., Pemberton, J. M. et al. (2003) Identification and in vivo characterization of PpaA, a regulator of photosystem formation in *Rhodobacter sphaeroides*. Microbiology 149: 377-388.

^3^ Zeller, T. & Klug, G. (2004) Detoxification of hydrogen peroxide and expression of catalase genes in *Rhodobacter*. Microbiology 150: 3451-3462.
